# ?Addendum to a minimalist revision of Costa Rican Braconidae: 28 new species and 23 host records

**DOI:** 10.3897/zookeys.1075.72197

**Published:** 2021-12-07

**Authors:** Michael J. Sharkey, Austin Baker, Kathryn McCluskey, Alex Smith, Suresh Naik, Sujeevan Ratnasingham, Ramya Manjunath, Kate Perez, Jayme Sones, Michelle D’Souza, Brianne St. Jacques, Paul Hebert, Winnie Hallwachs, Daniel Janzen

**Affiliations:** 1 The Hymenoptera Institute, 116 Franklin Ave., Redlands, CA, 92373, USA The Hymenoptera Institute Redlands United States of America; 2 Department of Entomology, University of California, Riverside, CA 92521, USA University of California Riverside United States of America; 3 Department of Biology, University of Pennsylvania, Philadelphia, PA 19104-6018, USA University of Pennsylvania Philadelphia United States of America; 4 Department of Integrative Biology, University of Guelph and Biodiversity Institute of Ontario, Guelph, Canada University of Guelph and Biodiversity Institute of Ontario Guelph Canada; 5 Centre for Biodiversity Genomics, University of Guelph, Guelph, Canada University of Guelph Guelph Canada

**Keywords:** Accelerated taxonomy, BIN code, conservation, COI DNA barcode, Hymenoptera, Ichneumonoidea, parasitoid host associations, tri-trophic interaction.

## Abstract

Twenty-nine species are treated, most of which have host caterpillar and food plant records, and all but one are new to science. The first host record for the agathidine genus *Amputoearinus* is given. *Gnathopleurajosequesadai* Sharkey, **sp. nov.** is reported as a hyperparasitoid of fly larvae, the first such record for the genus. The following new species are diagnosed primarily using COI barcode data; Sharkey is the authority for all: Agathidinae: *Aerophilusdavidwagneri, Aerophilusfundacionbandorum, Aerophilusnicklaphami, Lytopylusdavidstopaki, Lytopylusdavidschindeli*; Alysiinae: *Gnathopleurajosequesadai*; Braconinae: *Braconandreamezae, Braconfranklinpaniaguai, Braconrafagutierrezi, Braconguillermoblancoi, Braconoscarmasisi, Braconpauldimaurai, Braconshebadimaurae, Saciremakarendimaurae*; Cheloninae: *Chelonusminorzunigai*; Homolobinae: *Homolobusstevestroudi*; Macrocentrinae: *Macrocentrusmichaelstroudi*; Orgilinae: *Stantoniagilbertfuentesi*; Rhysipolinae: *Rhysipolisstevearonsoni*; Rogadinae: *Aleiodeskaydodgeae, Aleiodeskerrydresslerae, Aleiodesjosesolanoi, Aleiodesjuniorporrasi, Aleiodesrocioecheverri, Aleiodesronaldzunigai, Choreborogasjesseausubeli, Triraphisdoncombi*, and *Yeliconesmayrabonillae*.

## ?Introduction

The purpose of this research is to diagnose and name 28 new species of Costa Rican braconids. We deal with braconid subfamilies: Agathidinae, Braconinae, Cheloninae, Macrocentrinae, Orgilinae, Proteropinae, Rhysipolinae, and Rogadinae. Of these 28 species, 23 are reared from host caterpillars and their food plants are documented. This is an addendum to Sharkey et al. (2021a) where 403 species were diagnosed by using COI DNA barcode sequences. Zamani et al. (2020) criticized our barcoding approach and responses to their criticisms can be found in Sharkey et al. (2021a and 2021b).

We briefly describe the fate of large monographs that treat small portions of hyper-diverse Neotropical ichneumonoids by exemplifying the Sharkey (1988) revision of *Alabagrus* (Braconidae) and the Dasch (1974) revision of *Mesochorus* (Ichneumonidae). We emphasize that in these two publications the keys and descriptions required enormous time, effort, and expense; they are little used; and when they are used, the results are usually erroneous. This makes them useless or even detrimental to the taxonomy and understanding of these groups.

According to a search in Google Scholar (May 2021), the *Alabagrus* revision has 32 citations and the *Mesochorus* revision has 39. The majority of the citations are geographical surveys that simply copy the distributional records that are in these papers. For example, Rodríguez-Berrío et al. (2009) surveyed the literature for all Ichneumonidae occurring in Peru and included a number of species cited as being present in Peru by Dasch (1974); the keys and descriptions were not employed.

Only four publications dealing with Neotropical *Alabagrus* employed the key of Sharkey (1988); in three of these cases the specimens were identified by or checked by Sharkey himself. The quality of the identifications in one of these, Leathers and Sharkey (2003), was reported in Sharkey et al. (2018, 2021a), and our self-criticisms are repeated here. Leathers and Sharkey (2003) used the key and also had access to identified specimens. Nonetheless, of the 17 species that they reported to occur in Costa Rica, none are now realized to occur in Costa Rica because they only live elsewhere, and Costa Rican undescribed species were mistaken for them. These are *Alabagrus albispina, A. imitatus, A. juchuy, A. kagaba, A. latisoma, A. latreillei, A. maya, A. mojos, A. nahuatl, A. nigrilitus, A. pachamama, A. paruyana, A. parvifaciatus, A. semialbus , A. tricarainatus, A. tripartitus*, and *A.warrau*. Furthermore, five of the species reported by Leathers and Sharkey (2003) were found to be composed of species complexes as determined by a combination of morphological, barcode, and host data. These five are *Alabagrus cocto, A. englishi, A.pecki, A.roibasi, and A. yaruro* (Sharkey et al. 2018). There is no reason to believe that the other two publications in which Sharkey played a role in identification of specimens of *Alabagrus* (Braet 2002, Cauich-Kumul 2012) are any better, despite his being the world authority on identification of Agathidinae. The sole publication in which Sharkey did not play a role in identification was one dealing with the Brazilian fauna (Yamada et al. 2006). In this publication 21 species of *Alabagrus* were identified. Of these, ten of the holotypes are from Mexico or Central America and one is from the United States. The likelihood of any of these occurring in Brazil is extremely low and yet they probably fit the key in Sharkey (1988). We estimate there to be many more than 1,000 species of *Alabagrus* in the Americas; the probability of the undescribed species fitting the key is therefore obviously high. A key that deals with only 10% of the fauna is all but useless, and if all of the species were described, the key would be more than 1,000 couplets long; impossible to work with. A key of this length would preclude accurate identifications due to user error or location inaccuracies (e.g., a Brazilian specimen that looks like a Mexican specimen has a high probability of being a different species).

In summary, in the 30-plus years since the publication of the morphology-based revision of *Alabagrus*, only one person other than Sharkey has used the key to arrive at a determination for Neotropical species, and in that instance most of the identifications are probably incorrect. It took Sharkey more seven years to produce the 1988 revision, and it is worse than useless because it is full of misleading information on species limits and species distributions, owing to misidentifications. Some might argue for an integrative approach, such as the revision of *Alabagrus* by Sharkey et al. (2018), but what is the point of including morphological descriptions, which tend to be lengthy and time-consuming to produce, when the COI barcode is the only reliable source for identification (barring much more expensive and complex multi-gene information)?

There are many genera of ichneumonoids that contain hundreds or thousands of species in the Neotropics, but few of these have been revised for the entire area. One of these exceptions, besides Sharkey (1988), is the revision of Neotropical *Mesochorus* (Dasch 1974), a genus of hyperparasitoid Ichneumonidae. Dasch treated what he considered to be 245 Neotropical species, and like the Sharkey revision of *Alabagrus*, few publications have used his keys or descriptions to identify specimens of *Mesochorus*; we have located three. Of all of the 245 species of Neotropical *Mesochorus* that he treated, 30 were recorded from Costa Rica. Based on the 172 BINs of Costa Rican *Mesochorus* presently on BOLD (March 2, 2021), we estimate that there are approximately 688 species in Costa Rica. These species are almost exclusively from the Area de Conservación Guanacaste, from rearings that have been conducted exclusively in the provinces of Guanacaste and Alajuela (Janzen and Hallwachs 2011). Given this estimate, the odds of a Costa Rican specimen being in Dasch’s (1974) key is 4.4%. The fact that these large revisions are not useful is not the result of poor workmanship, nor is it that the readers are poorly trained. In fact, the only users are highly trained taxonomists specializing in Ichneumonoidea. The keys are not used simply because they do not work. Not only are the species concepts poor with many species having similar morphology, but the revisions deal with such a small portion of the total number of species that the odds of a specimen in hand being in the key is remote.

## ?Materials and methods

### ?Delimiting species

We received considerable critical feedback after the publication of Sharkey et al. (2021a). One of the most common criticisms directed at our approach is that barcodes in general, and BINs in particular, are not capable of delimiting species. This was dealt with at length in Sharkey et al. (2021a); nonetheless in an effort to avoid further confusion, we describe in detail below the process we go through to arrive at species limits or at the least, species central tendencies. BIN is an abbreviation for Barcode Index Number and an article by Ratnasingham and Hebert (2013) describes how and why the BIN algorithm was developed. They describe the BIN system as a means of forming Operational Taxonomic Units (OTUs) based on divergence in COI sequences. In essence, the BIN is like a unit tray of specimens believed to be monospecific by similarity of contained barcodes rather than appearance. They clearly state that no system like this can be perfect, “Any algorithmic approach based on the analysis of sequence diversity in a single gene region will be an imperfect tool for the discrimination of closely related species as they will be overlooked because of their low sequence divergence.” (Ratnasingham and Hebert 2013: 2).

We start a revision by grouping our specimens into unit trays based on their BIN placements. The specimens in each tray are then investigated for general morphological consistency, and inconsistent specimens are flagged. This is followed by an inspection of a NJ tree that we generate on the BOLD website using only those specimens with full or almost full barcodes, i.e., barcodes with 500–658 base pairs. We carefully examine the branching pattern of the specimens in each BIN. If there is any clumping or any outliers in the tree of the specimens within a BIN, we look at the rearing host data and microgeography, if it is available. We also look at the morphology of the specimens, to see if they differ significantly and check for concordance between these three data sources. If these data sources are consistent with the hypothesis that any cluster of branches represents a separate species within the BIN, we consider this possibility based on the degree of difference in morphology, sequence divergence, and host use. We then build a new NJ tree that includes shorter barcodes to place those specimens into species formulated in the previous step and to add new species that may not be represented by specimens with full barcodes. Finally, we look at the morphology and host data of the nearest neighbors for each BIN. If these do not differ morphologically, we might consider this to be a case where a pair of BINs split a species. This, of course, depends on the degree of COI divergence. To date, we have found no such case. Specimens that fail to barcode, are contaminated, or are otherwise not barcodeable, are excluded from consideration, but the specimen and its record are retained. There are times when a reared specimen is obviously conspecific with others reared from the same host species but not currently barcodeable, and therefore, they are only retained for ecological analyses, such as what fraction of caterpillars were killed by that parasitoid.

Co-author Janzen estimates that the BIN algorithm lumps two or more sympatric species of Costa Rican Lepidoptera within a BIN at a rate of ~ 10%. And in those cases, almost invariably the multiple species are evident by genitalic differences, caterpillar food plant, microgeography, and/or extremely slight differences in coloration. He also has not come across a case in which a species is split into more than one BIN, although this is certainly possible through within-species barcode polymorphisms. Thus, the BIN algorithm can be described as conservative. The 403 new species in Sharkey et al. (2021a) were grouped into 395 BINs with only three “multi-species” BINs, for a 2% BIN “error” rate. Error is in quotes here because the barcodes do separate the species, but the BINs do not in these few cases.

In the following paragraphs we give an empirical example of how we arrive at species delimitations; we do not say “species limits” because these geobiological limits have not been explored further than ACG, or Costa Rica, or the Neotropics. BIN BOLD:ACK7466, treated in Sharkey et al. (2021a), is a BIN with multiple species, and there are also a handful of examples in the literature (e.g., Hebert et al. 2004; Janzen et al. 2017). In this BIN, BOLD:ACK7466, we have what are probably 11 species, nine of which we have described, and one is in the current publication. Each of these 11 species matches a distinctive set of host caterpillars yet are fully sympatric, just as was the case for 6 of the first 11 sympatric species found to be described as a single species, *Astraptesfulgerator* (Hesperiidae) (Hebert et al. 2004). To help discover potential species within a BIN, the first NJ tree that we build employs only those sequences with complete or almost complete barcodes, e.g., > 500bp. The portion of the tree that contains the specimens of BIN BOLD:ACK7466 is presented in Figure 1. The reason for using almost complete barcodes at this stage is to base our decisions on the highest quality data. A quick look at the tree (Fig. 1) shows that there are a number of specimens with identical sequences that cluster together on different branches of the tree. We investigate each cluster individually. For example, the two specimens of *M.michaelstroudi* sp. nov. (branch A at the top of the tree in Figure 1) are consistent with the hypothesis of being a separate species because: 1. They have the same barcode sequence, which is quite divergent from other members of the clade. 2. Their hosts are the same crambid, *Phaedropsis* leialisDHJ03, and no other specimens in the BIN attack members of this genus. 3. These two specimens are morphologically different from all others in the BIN, the details of which are in the diagnosis in the species treatment; “In the morphological key for the species in this BIN, *Macrocentrusmichaelstroudi* keys to *M.gustavogutierrezi*. *Macrocentrusmichaelstroudi* differs in having pale basal flagellomeres, contrasting with the melanic basal flagellomeres of *M.gustavogutierrezi* (Sharkey et al. 2021a).

**Figure 1.**
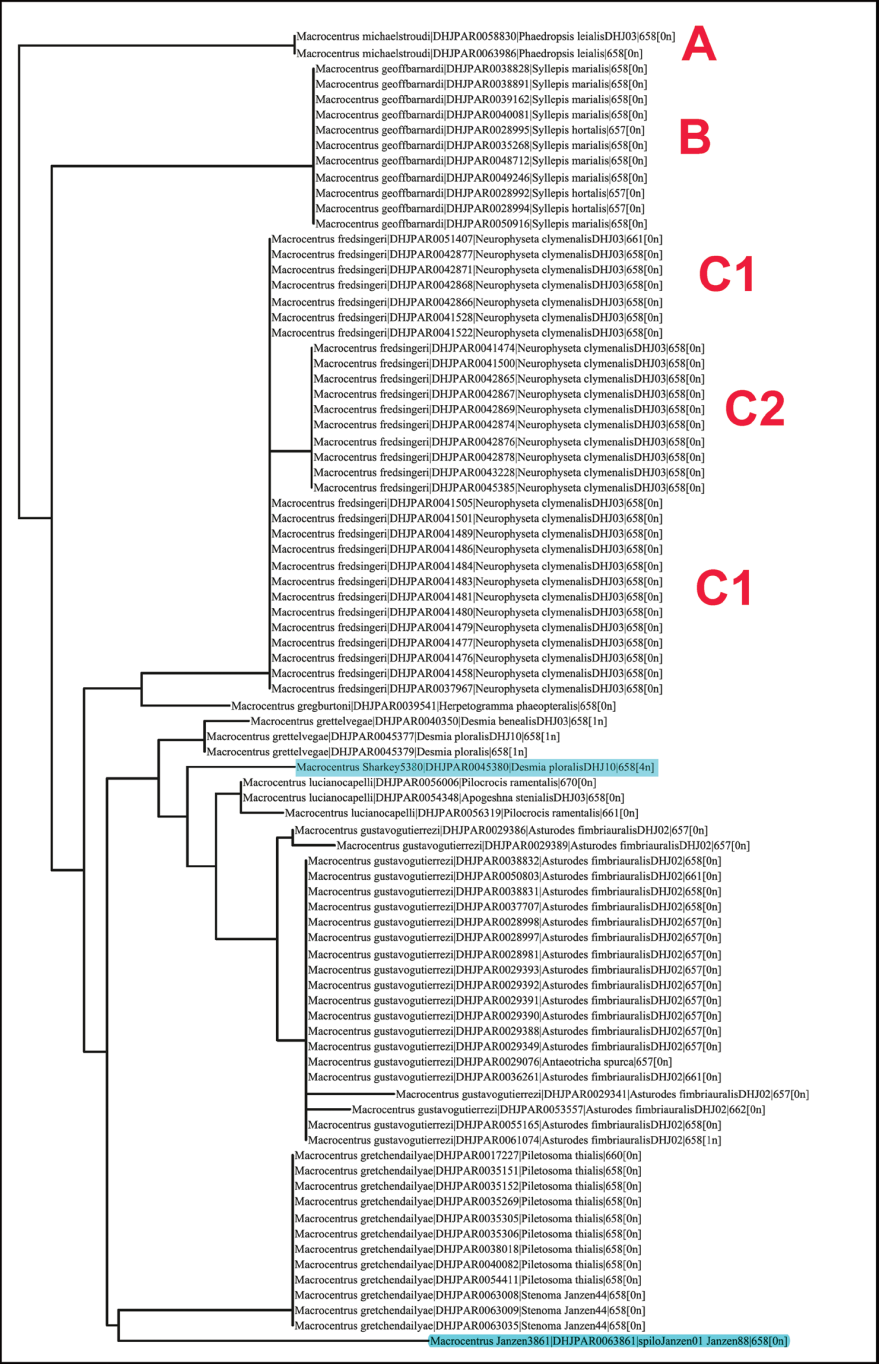
NJ tree of *Macrocentrus* BIN BOLD:ACK7466.

Within the cluster of specimens identified as *Macrocentrusgeoffbarnardi* (Fig. 1, branch B) we have the same situation as in *M.michaelstroudi*, so we compare with the specimens on the branch with specimens of *M.fredsingeri* (Fig. 1, branch C). Here we have two clusters (C1 and C2) that are joined on a relatively long branch. Members of branches C1 and C2 are all parasitoids of *Neurophysetaclymenalis*DHJ03. (*N.clymenalis*DHJ03 is the interim name for a species, probably unnamed, that is similar to *N.clymenalis*). The specimens on C1 cannot be separated from those on C2 on morphological grounds. However, the entity (C1 + C2) can be separated on morphological grounds from all of the other specimens in BIN BOLD:ACK7466. Finally, no other specimens in the BIN are parasitoids of species of *Neurophyseta*. Therefore, we considered the entire cluster (C1 + C2) as one species. If further examination or data suggest that it is two, then one more will be also described. Similar arguments were used to delimit the other nine species in the BIN (Fig. 1). The specimens highlighted in blue (Fig. 1) represent probable new species that have not yet crossed the desk of author Sharkey because they are still in the barcoding pipeline.

The next step in our process is to add specimens to the analysis with less COI data, i.e., shorter COI barcodes. This often produces a “noisier” NJ tree. Here we just show a small segment of the NJ tree to make our point (Fig. 2). The highlighted terminals have shorter barcodes and are new to the tree of Figure 1. Neither falls in the large homogeneous polytomy of *M.gustavogutierrezi*, and this is not uncommon for specimens with shorter barcodes. Specimens of this sort are looked at more carefully both morphologically and biologically and may or may not be included in the paratype series. In this case the two specimens share the same host, which is unique to the species, and do not differ morphologically in a substantive way.

**Figure 2.**
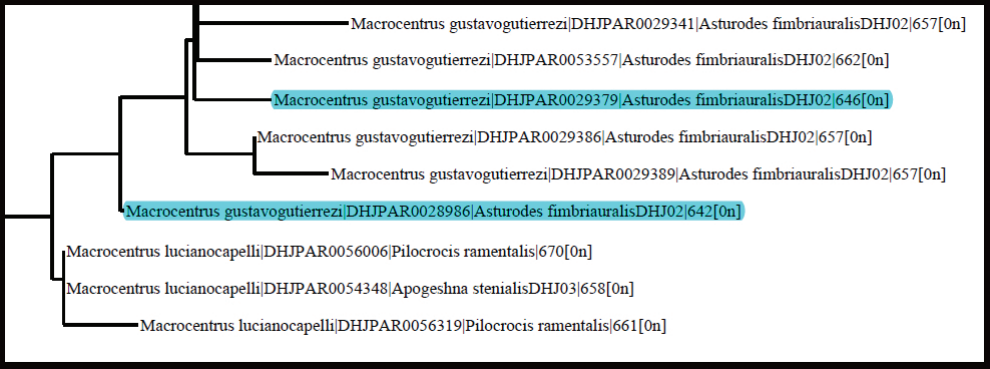
Portion of the NJ tree generated from BOLD showing additions of sequences with barcodes shorter than 650bp. These are highlighted in blue.

By this stage we have examined the membership of each BIN to determine whether there is one or more species in the BIN. The final step before species description is to investigate the nearest neighbor of each BIN to ensure that they differ morphologically and/or biologically. To date, all BINs examined for Braconidae have differed from their neighboring BINs. The nearest neighbor can be found on the BOLD website. For example, to find the nearest neighbor of BIN BOLD:ACK6477, we search for that BIN on the BOLD database and are taken to a page that includes the information in Figure 3. In this case the nearest neighbor to BIN:BOLD:ACK6477 is *Macrocentrusiangauldi*, which occupies BIN BOLD:ABY7812. Specimens in the two BINs are then compared to ensure that they differ morphologically and biologically, which they do. We stated earlier that the BIN algorithm failed to be mono-specific at a rate of 2% in the treatment by Sharkey et al. (2021a), but it is worth noting that the barcode sequences themselves did not fail. Even when there are multiple species within a BIN, the COI sequences differentiated the included species as seen in Figure 1; these results are corroborated by host data and morphology.

**Figure 3.**
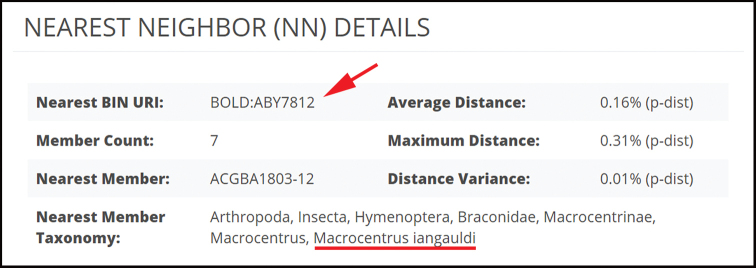
A portion of the webpage on BOLD for BIN BOLD:ACK7466.

In contrast to COI barcode diagnostics, we have found cases in which morphology cannot discriminate species that are clearly diagnosed by COI barcodes. Of the 86 species treated in the revision of *Alabagrus* by Sharkey et al. (2018) there were three species that could not be separated morphologically but were clearly delimited based on host data and COI sequence data. The final couplet for this group from the key by Sharkey et al. (2018) is as follows:

**Table:** 

**102 A**	Forewing with yellowish or clear area extending to the 2^nd^ submarginal cell	***A.roibasi***
**102 B**	Forewing entirely infuscate, or if with yellowish or clear area basally, it does not extend to 2^nd^ submarginal cell (we cannot distinguish the following 3 species morphologically)	***A.jennyphillipsae; A.isidrochaconi; A.jeanfrancoislandryi***

Figure 4 is a portion of the tree of highest posterior probability (from Sharkey et al. 2018) based on COI data showing the relationships of these three species (indicated with a red dot). The NJ tree produced by BOLD indicates slightly different relationships but, as with the Bayesian tree, the three morphologically indistinguishable species are not sister species nor are they nearest neighbors by any definition of that concept. We may have made different decisions if these lineages shared hosts or formed a monophyletic clade and were represented by very few sequences.

**Figure 4.**
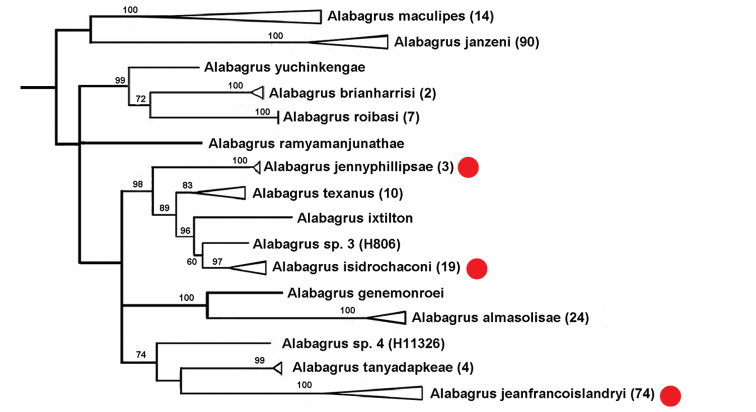
Portion of the tree of highest posterior probability from a 10 million-generation Bayes analysis of COI. The numbers of specimens of each species are collapsed (when possible) into single terminals (terminal triangles), with the number of specimens/OTUs for each collapsed species in parentheses. The length of the triangles represents the branch length from the node to the tip of the longest branch for that species. The numbers above the branches are the posterior probabilities × 100. The red dots indicate the three species that could not be differentiated morphologically. Modified from Sharkey et al. (2018).

### ?Specimens and generic placements

As with those of Sharkey et al. (2021a), most of the species described here were collected by rearing wild-caught host caterpillars in ACG in northwestern Costa Rica (Janzen and Hallwachs 2016). Holotypes of all newly described species are deposited in the insect collection of the Canadian National Collection of Insects, Ottawa. Paratypes and all other specimens are currently deposited in the Centre for Biodiversity Genomics in the Biodiversity Institute of Ontario at the University of Guelph.

Identification of specimens to the subfamily level can be achieved using the key by Sharkey (1997). Keys to the genera of the species treated here are found in Sharkey et al. (2021a) and references therein. However, identification to any level is best acquired by obtaining COI barcodes and submitting them to BOLD. Instructions on how to do this are included below.

Some host species treated here are awaiting full identification and are given interim names. For example, *Antaeotricha* Janzen233 is identified to the genus *Antaeotricha* by classical morphology-based criteria and to Janzen233 by barcode and ecological information. However, no formal scientific species name is available until a barcode-match is obtained with an existing holotype or until it is described as new, or interim matched morphologically with a described species by a taxonomic specialist, which may take decades. Equally, Antaeotricharadicalis EPR03 is also an interim name based on what the species looks like, however, it is not a scientific name. It temporarily retains the information that this species is recognized by similarity with its look-alike, *A.radicalis*, before barcoding and associating it with other ecological data. Finally, a name such as gelJanzen01 Janzen407 signifies a caterpillar in the family Gelechiidae for which even a generic name is not obtainable at present.

### ?DNA extraction and sequencing

Molecular work was carried out at the CBG using their standard protocols. A leg of each frozen-then-oven-dried specimen was destructively sampled for DNA extraction using a glass fiber protocol (Ivanova et al. 2006). Extracted DNA was amplified for a 658-bp region near the 5' terminus of the cytochrome *c* oxidase subunit I (COI) gene using standard insect primers LepF1 (5'-ATTCAACCAATCATAAAGATATTGG-3') and LepR1 (5'-TAAACTTCTGGATGTCCAAAAAATCA-3') (Ivanova and Grainger 2007). If initial amplification failed, additional amplifications were conducted following the established protocols using internal primer pairs: LepF1–C113R (130 bp) or LepF1–C_ANTMR1D (307 bp) and MLepF1–LepR1 (407 bp) to generate shorter overlapping sequences. Amplified products were sequenced using Sanger technology, though the most recent were sequenced by SEQUEL II. Specimens that “failed” barcoding are not included here unless otherwise indicated. When included, they are usually identified by unambiguous morphological and ecological information equally possessed by others from ACG in that species.

Barcode sequences presented in the species descriptions herein are a consensus of the barcode sequences of all included individuals, meaning base pairs that differ between conspecific specimens are replaced by IUPAC ambiguity codes.

### ?Databases

Voucher codes are presented for all holotype specimens (and all other barcoded individuals) treated herein. All host caterpillars are individually vouchered to their individual records (yy-SRNP-xxxxx). Codes beginning with DHJPARxxxxxxx are for the parasite (or hyperparasite) specimens reared from the caterpillar; therefore, each wasp carries two voucher codes, one for the rearing (host) record and one for the wasp itself. The SRNP voucher codes are from the Janzen and Hallwachs’ database (http://janzen.sas.upenn.edu/caterpillars/database.lasso). Specimen voucher codes beginning with BIOUG are from the BOLD database (http://www.boldsystems.org), and most of the specimens obtained from ACG Malaise traps have this prefix. The DHJPAR and their associated SRNP codes can also be found on the BOLD database. The abundant collateral information obtainable from these two databases complements the species treatments. See Sharkey et al. (2021a) for a brief introduction to what to look for and how the two databases supplement the species treatments herein.

The BOLD database can be used to identify specimens using the following steps: 1. Navigate to the identification tab of the BOLD Systems database (http://www.boldsystems.org/index.php/IDS_OpenIdEngine). 2. Paste the COI sequence of the query organism (in forward orientation) into the query box and search against the appropriate library (e.g., All Barcode Records on BOLD, Species Level Barcode Records, etc.). 3. The search results page shows the top hits based on % similarity starting with the closest matches. This page also provides additional information to help verify the identity of a match, such as links to the BIN where specimen data (including images) can be found, a distribution map, and a tree-based identification tool. 4. Use the Tree-Based Identification button to generate a neighbor-joining tree and find the query taxon (name in red). This allows you to visualize how distant the query sequence is from the closest matches.

## ?Taxonomic account

### ?Agathidinae

A key to the genera of the New World can be found in Sharkey et al. (2021a). Agathidines are cosmopolitan and exclusively koinobiont endoparasitoids of caterpillars. They emerge from the host after the caterpillar is full-grown and has begun to spin or has already spun a cocoon.

#### ? Aerophilus davidwagneri

Taxon classificationAnimaliaHymenopteraBraconidae

Sharkey sp. nov.

4949F5EF-3CDD-55B0-BB0E-E1FF40444BC8

http://zoobank.org/DA927AD6-EB95-45B2-8570-F29BB42F9968

##### Diagnostics.

Figure 5.

##### BOLD data.

BIN: BOLD:ACJ2677; nearest neighbor: *Aerophilusbradzlotnicki*BOLD:ACA4771; distance to nearest neighbor is 3.9%. Consensus barcode: AATTTTATATTTTATTTTTGGAATTTGAGCAGGAATTGTAGGATTATCAATAAGAATAATAATTCGAATAGAATTAAGAATAGTAGGTAATTTAATTGGTAATGATCAAATTTATAATAGAATTGTTCTGCTCATGCTTTTGTAATAATTTTTTTTATAGTTATACCAATTATAATTGGAGGATTTGGTAATTGATTAGTACCCTTAATATTAGGAGGTCCTGATATAGCTTTTCCTCGAATAAATAATATRAGATTTTGATTATTAATTCCTTCATTATTATTATTAATTTTAAGATCTTTARTTAATATTGGTGTAGGTACTGGATGAACTGTTTACCCTCCTTTATCATTAAATATAAGACATAATGGAATATCAGTAGATTTAGCTATTTTTTCTTTACATATTGCAGGTATTTCATCAATTATAGGAGCAATTAATTTTATTACAACTATTATAAATATATGAATAATTAATGTAAAAATTGATAAAATACCTTTAATAATTTGATCAATTTTTATTCTGCTATTTTATTATTATTATCTTTACCTGTTTTAGCTGGTGCTATT-CTATATTATTAACTGATCGAAATTTAAATACTAGATTTTTTGATCCTACAGGAGGAGGAGATCCAATTTTATATCAACATTTATTT.

##### Morphological data.

This species can be morphologically distinguished from its nearest neighbor by having the mesosoma entirely black (Fig. 5) compared to having large orange patches on the lateral sides (Sharkey et al. 2011: figs 3, 4).

**Figure 5.**
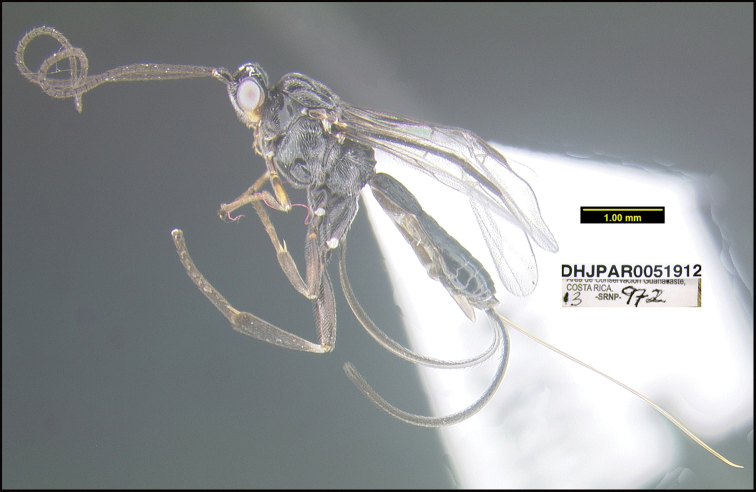
*Aerophilusdavidwagneri*, holotype.

**Holotype** ?: Costa Rica: Alajuela, Area de Conservación Guanacaste, Sector San Cristobal, Cementerio Viejo, 570 m, 10.88111 -85.38889; host caterpillar collection date: 27/ii/2013, parasitoid eclosion: 19/iii/2013; depository CNC, holotype voucher code: DHJPAR0051912.

##### Holotype host data.

*Polyortha* Janzen226 (Tortricidae) feeding on *Desmopsisschippii* (Annonaceae). Host caterpillar voucher code13-SRNP-972

##### Paratype.

Hosts are all the same as that of the holotype: DHJPAR0054734, DHJPAR0055235, DHJPAR0051139, DHJPAR0051915, DHJPAR0055516, DHJPAR0054741, DHJPAR0055237, DHJPAR0054728, DHJPAR0055233.

##### Etymology.

*Aerophilusdavidwagneri* is named in honor of David Wagner of the University of Connecticut, Storrs, Connecticut, USA, for his recent work as an environmental activist for a healthier global climate and wild biodiversity.

#### ? Aerophilus fundacionbandorum

Taxon classificationAnimaliaHymenopteraBraconidae

Sharkey sp. nov.

EBD717AC-52CC-5638-BAA9-AA8747A70927

http://zoobank.org/A73266E4-1332-4185-816A-4AD9EEDACEC4

##### Diagnostics.

Figure 6.

##### BOLD data.

BIN: BOLD:ACN0950; nearest neighbor: *Aerophiluscalcaratus*BOLD:AAU4711; distance to nearest neighbor is 5.81%. Consensus barcode AATTTTATATTTTATTTTTGGAATTTGATCTGGTATTTTAGGATTATCAATAAGAATCATTATTCGTATAGAATTAAGATTAGGGGGTAATTTAATTGGTAATGATCAAATTTATAATAGAATTGTTCTGCTCATGCTTTTGTAATAATTTTTTTTATAGTTATACCGATTATAATTGGAGGATTTGGAAATTGATTAGTTCCTTTAATGTTAGGAGGTCCAGATATGGCCTTTCCACGRATAAATAATATAAGATTTTGATTATTAATTCCTTCATTAACTTTATTAATTTTAAGATCAATATTAAATGTTGGTGTAGGTACGGGATGAACTGTYTATCCTCCCTTATCATTAAATATAAGTCATAGAGGAATATCTGTAGATTTAGCAATTTTTTCTTTACATAYTGCTGGAATTTCTTCTATTATAGGAGCAATAAATTTTATTACTACAATTATTAATATATGRATAATAAATGTAAAAATTGATAAAATACCTTTATTGGTATGATCTATTTTTATTCTGCTATTTTATTATTATTATCTTTACCAGTATTAGCTGGGGCTATTCTATATTATTAACTGATCGAAATTTAAATCTAGATTTTTTGATCCTTCTGGAGGAGGAGATCCAATTTTATATCAACACTTATTT

##### Morphological data.

*Aerophilusfundacionbandorum* keys to *A.macadamiae* in Sharkey et al. (2011). *Aerophilusfundacionbandorum* differs in many ways. One of the most obvious is its wide, sharply angled, median propodeal areola (Fig. 6). In *A.macadamiae* the areola is gradually narrowed anteriorly. *A.fundacionbandorum* can be morphologically distinguished from its nearest neighbor, *A.calcaratus*, by its more heavily sculptured first metasomal tergum. It is mostly striate in *A.fundacionbandorum* and mostly smooth in *A.calcaratus* (Sharkey et al. 2016: figs 12, 13).

**Figure 6.**
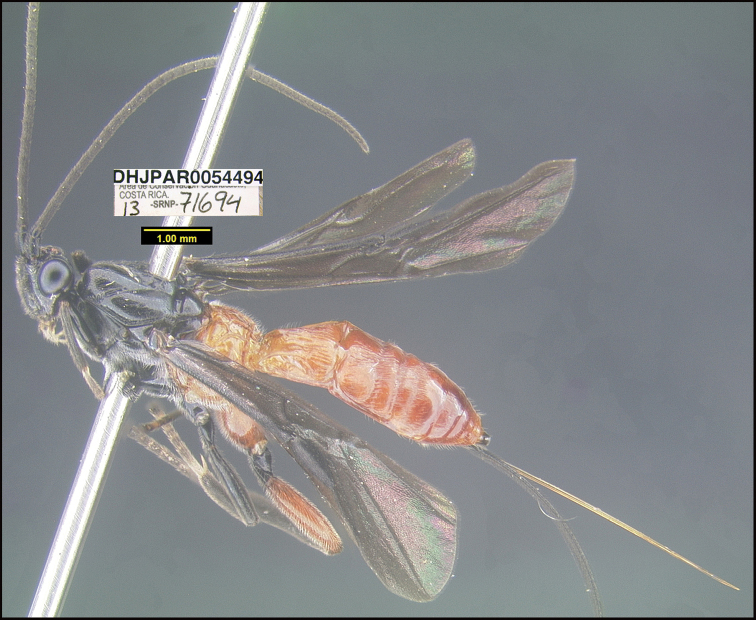
*Aerophilusfundacionbandorum*, holotype.

**Holotype** ?: Costa Rica: Guanacaste, Area de Conservación Guanacaste, Sector Pitilla Bullas, 440 m, 10.98670 -85.38503; host caterpillar collection date:07/x/2013, parasitoid eclosion: 12/xi/2013; depository CNC, holotype voucher code: DHJPAR0054494.

##### Holotype host data.

*Loxiorhizaunitula* (Thyrididae) feeding on *Schnellaguianensis* (Fabaceae), caterpillar voucher code13-SRNP-71694.

##### Paratype.

Same host species as that of holotype DHJPAR0054547.

##### Etymology.

*Aerophilusfundacionbandorum* is named in honor of the BAND Foundation of the USA, in recognition of its decades of support for growth and development of Costa Rica’s Área de Conservación Guanacaste and most recently for adding 85 more hectares to ACG of original forest, Bosque Transición, lying on the nearly extinct fusion of dry forest with rain forest (http://www.gdfcf.org/content/introducing-bosque-transición).

#### ? Aerophilus nicklaphami

Taxon classificationAnimaliaHymenopteraBraconidae

Sharkey sp. nov.

7B32CE61-1022-5440-8680-A2BCE450C6AE

http://zoobank.org/5C52FDA0-10E1-4152-9EE5-4D7BFE64516E

##### Diagnostics.

Figure 7.

##### BOLD data.

BIN: BOLD:ACT7814; nearest neighbor: *Aerophiluscolleenhitchcockae*BOLD:ACA4890; distance to nearest neighbor is 5.16%. Consensus barcode: AATTTTATATTTTATTTTTGGAATTTGATCTGGAATTTTAGGATTATCAATAAGAATAATTATTCGTATAGAATTAAGATTAAGGGGCAATTTAATTGGAAATGATCAAATTTATAATAGAGTTGTT-CTGCTCATGCTTTTGTTATAATTTTTTTTATAGTTATACCAATTATGATTGGGGGTTTTGGTAATTGATTAATTCCTTTAATATTAGGAGGTCCAGATATAGCATTTCCTCGTATAAATAATATAAGATTTTGATTATTAATTCCTTCTTTATTTTTATTAATTTTAAGTTCAATATTAAATATTGGAGTAGGTACAGGATGAACTGTTTATCCTCCTTTATCATTAAATATAAGACACAGAGGAATATCTGTAGATTTAGCAATTTTTTCTTTACATATTGCTGGAATTTCTTCTATTATAGGGGCAATAAATTTTATTACTACAATTATTAATATATGAATAATAAACGTAAAAATTGATAAAATACCTTTATTAGTATGATCCATTTTTATT-CTGCTATTTTATTATTATTATCTTTACCAGTATTGGCTGGAGCTATT-CTATATTATTAACAGATCGAAATTTAAAT-CTAGATTCTTTGATCCTTCAGGGGGAGGAGATCCTATTTTATATCAACATTTATTT

##### Morphological data.

This species keys to *A.jessicadimauroae* in Sharkey et al. (2011), but *A.nicklaphami* differs in many ways. Two of the most obvious are the wide sharply angled median propodeal areola and the sharp lateral longitudinal carinae on the first metasomal median tergite. In *A.jessicadimauroae* the areola is gradually narrowed anteriorly and the carinae are not sharp.

This species can be morphologically distinguished from its nearest neighbor, *Aerophiluscolleenhitchcockae*, by having the hind coxa and femur entirely brown (Fig. 7) compared to mostly black (Sharkey et al. 2011: figs 5, 6).

**Figure 7.**
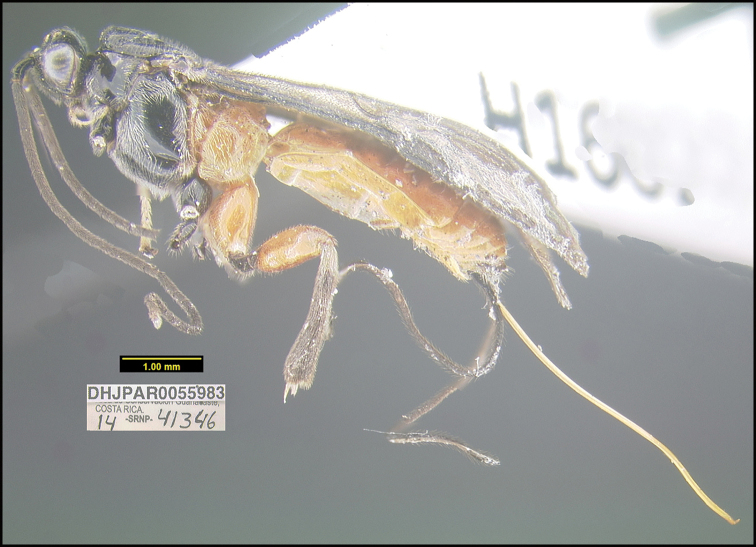
*Aerophilusnicklaphami*, holotype.

**Holotype** ?: Costa Rica: Alajuela, Area de Conservación Guanacaste, Sector Rincon Rain Forest Sendero Anonas, 405 m, 10.90527 -85.27881; host caterpillar collection date: 18/iii/2014, parasitoid eclosion: 02/v/2014; depository CNC, holotype voucher code: DHJPAR0055983.

##### Holotype host data.

*Tebenna* Janzen02 (Choreutidae) feeding on *Ficuscitrifolia* (Moraceae), caterpillar voucher code: 14-SRNP-41346.

##### Etymology.

*Aerophilusnicklaphami* is named in honor of Nick Lapham of the BAND Foundation of the USA, in recognition of his decades of support for growth and development of Costa Rica’s Área de Conservación Guanacaste, Costa Rica, and most recently adding 85 more hectares to ACG of original forest, Bosque Transición, lying on the nearly extinct fusion of dry forest with rain forest (http://www.gdfcf.org/content/introducing-bosque-transición).

#### ? Amputoearinus alafumidus

Taxon classificationAnimaliaHymenopteraBraconidae

Lindsay & Sharkey, 2006

D52030DC-E128-5B95-8D89-CA6C069F82ED

##### Diagnostics.

Figure 8.

##### BOLD data.

There is no BIN for this specimen because the barcode is too short to merit a BIN code. The short barcode follows:

ATATTTATTTAATTTTTGGAATTTGATCAGG-ATTTTAGGATTATCAATAAGAATAATTATTCGTATAGAATTAAGAATGGGGGGAAATTTTATTGGTAATGATCAAATTTATAATAGAATTGTT-CTGCTCATGCATTTATTATAATTTTTTTTAAAGTTATACCAATTATAATTGGAGGATTTGGAAATTGATTAATTCCTTTAATATTAGGGGGCCCAGAAAAAGCTTTCCCCCGAATAAATAATATAAAATTTTGAT

##### Morphological data.

This specimen was identified based on morphological criteria from the key in Lindsay and Sharkey (2006).

**Reared specimen**: ?: Costa Rica: Guanacaste, Area de Conservación Guanacaste, Sector Del Oro, Puente Mena, 280 m, 11.04562 -85.45742; host caterpillar collection date: 11/07/2007, parasitoid eclosion: 27/07/2007; depository CNC, voucher code: DHJPAR0028287.

**Reared specimens host data**: *Dysodiaspissicornis* (Thyrididae) a leaf-roller feeding on *Heisteriaconcinna* (Olacaceae), caterpillar voucher code: 07-SRNP-22487.

##### Note.

This is the first host record for this wasp genus.

**Figure 8.**
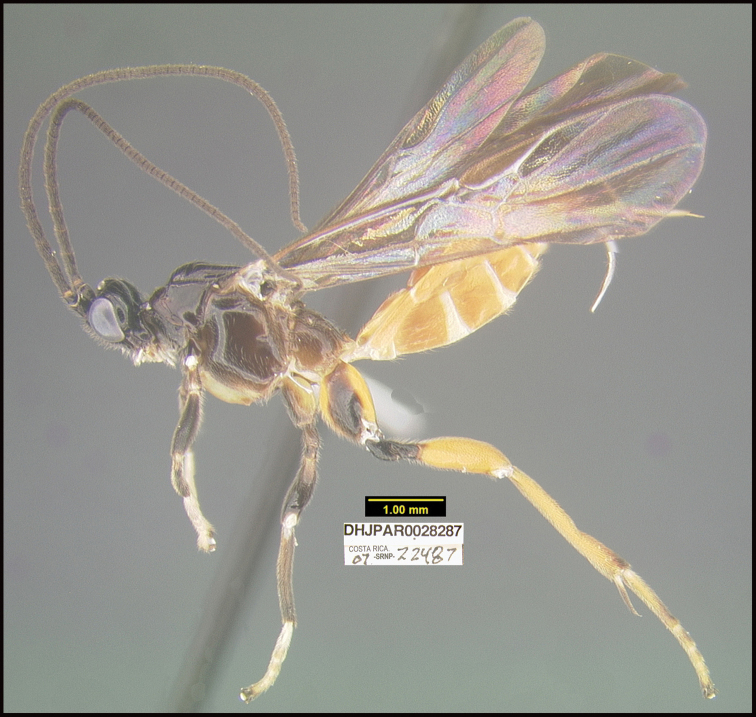
*Amputoearinusalafumidus*, holotype.

#### ? Lytopylus davidstopaki

Taxon classificationAnimaliaHymenopteraBraconidae

Sharkey sp. nov.

9AA5EF7E-2B85-5723-B06A-C334F012820B

http://zoobank.org/B88988CF-D2D5-4B57-ACC5-B3D5A8E7A2DF

##### Diagnostics.

Figure 9.

##### BOLD data.

BIN: BOLD:ACJ2185; nearest neighbor: *Lytopylusdavidschindeli*BOLD:ACB1289; distance to nearest neighbor is 2.56%. Consensus barcode: AATTTTATATTTTATATTTGGTATTTGATCAGGAATTTTAGGTTTATCATTAAGATTAATTATTCGAATAGAATTAAGAATTGGTGGAAATTTAATTGGAAATGATCAAATTTATAATAGAATTGTTACTGCTCATGCTTTTATTATAATTTTTTTTATAGTTATACCAATTATAATTGGAGGATTTGGTAATTGATTAATTCCTTTATTATTAGGAGGTCCTGATATAGCTTTCCCTCGAATAAATAATATAAGATTTTGATTATTAATTCCTTCATTATTATTATTAATTTTAAGATCTTTAATTAATATTGGTGTAGGTACAGGATGAACAGTTTATCCTCCATTATCTTTAAATATAAGTCATAGTGGTATATCTGTAGATATAGCAATTTTTTCTTTACATATTGCTGGAATTTCTTCAATTATAGGAGCTATAAATTTTATTACTACTATTATAAATATATGAATTTTAAATTTAAAATTTGATAAAATACCTTTATTAGTTTGATCAATTTTAATTACAGCAATTTTATTATTATTATCATTACCAGTTTTAGCTGGAGCTATTACTATATTATTAACTGATCGAAATTTAAATACAAGATTTTTTGATCCTTCAGGAGGAGGAGATCCAATTTTATATCAACATTTATTT

##### Morphological data.

This species keys to *L.youngcheae* in Kang et al. (2017) but differs in many ways. The easiest to see is that the hind coxae and bases of hind femora are partly black in *L.youngcheae* and entirely orange in *L.davidstopaki*. This species can be morphologically distinguished from its nearest neighbor, *Lytopylusdavidschindeli*, by having its mesosoma and coxae entirely tan (Fig. 9) compared to entirely black or dark brown (Fig. 10).

**Figure 9.**
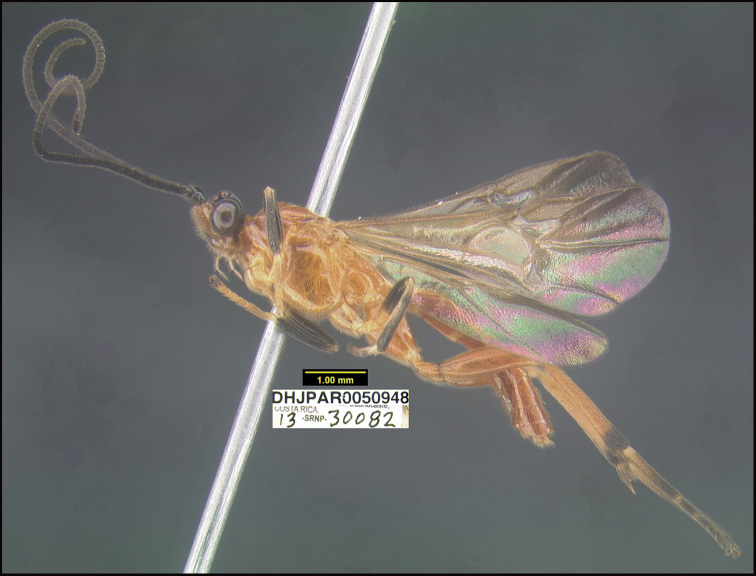
*Lytopylusdavidstopaki*, holotype.

**Holotype** ?: Costa Rica: Guanacaste, Area de Conservación Guanacaste, Sector Pitilla, Sendero Laguna, 680m, 10.9888 -85.42336; host caterpillar collection date: 10/i/2013, parasitoid eclosion: 01/ii/2013; depository CNC, holotype voucher code: DHJPAR0050948.

##### Holotype host data.

elachJanzen01 Janzen527 (Depressariidae) feeding on *Calatolacostaricensis* (Metteniusaceae), caterpillar voucher code:13-SRNP-30082.

##### Paratype.

Same host species as that of the holotype, DHJPAR0050946, DHJPAR0057411.

##### Etymology.

*Lytopylusdavidstopaki* is named in honor of David Stopak of the Editorial Office of the Proceedings of the National Academy of Sciences (PNAS), in recognition of his decades of editorial understanding and accepting the strange research emerging from the biodiversity inventory of Costa Rica’s Área de Conservación Guanacaste.

#### ? Lytopylus davidschindeli

Taxon classificationAnimaliaHymenopteraBraconidae

Sharkey sp. nov.

8C468F73-0A3F-557C-849D-396D447833BF

http://zoobank.org/5006423F-2393-4963-9EE1-8423DC2CE954

##### Diagnostics.

Figure 10.

##### BOLD data.

BIN: BOLD:ACB1289; nearest neighbor: *Lytopylusdavidstopaki*BOLD:ACJ2185; distance to nearest neighbor is 2.56%. Consensus barcode AATTTTATATTTTATATTTGGAATTTGATCAGGAATTTTAGGATTATCATTAAGATTAATTATTCGAATAGAATTAAGAATTGGAGGAAATTTAATTGGTAATGATCAAATTTATAACAGAATTGTAACTGCTCATGCTTTTATTATAATTTTTTTTATAGTTATACCAATTATAATTGGAGGATTTGGAAATTGATTAATTCCTTTAATATTAGGAGGTCCTGATATAGCTTTTCCTCGAATAAATAATATAAGATTTTGATTATTAATTCCTTCATTATTATTATTAATTTTAAGGTCTTTAATTAATATTGGAGTAGGAACAGGATGAACAGTTTATCCTCCTTTATCTTTAAATATAAGTCATAGTGGTATATCTGTAGATATGGCAATTTTTTCTTTACATATTGCTGGAATTTCTTCAATTATAGGAGCTATAAATTTTATTACTACTATTATAAATATATGAATTTTAAATTTAAAATTTGATAAAATACCTTTATTAATTTGATCAATTTTAATTACAGCAATTTTATTATTATTATCATTACCAGTTTTAGCTGGTGCTATTACTATATTATTAACTGATCGAAATTTAAATACAAGATTTTTTGATCCATCAGGAGGAGGAGATCCAATTTTATATCAACATTTATTT

##### Morphological data.

This species keys to *L.angelagonzalezae* in Kang et al. (2017) and differs in many respects. The most evident is that the propodeum of *Lytopylusdavidschindeli* is almost completely smooth with the central areola barely indicated. This species can be morphologically distinguished from its nearest neighbor, *Lytopylusdavidstopaki*, by having the mesosoma and coxae entirely black or dark brown (Fig. 10) compared to entirely tan (Fig. 9).

**Figure 10.**
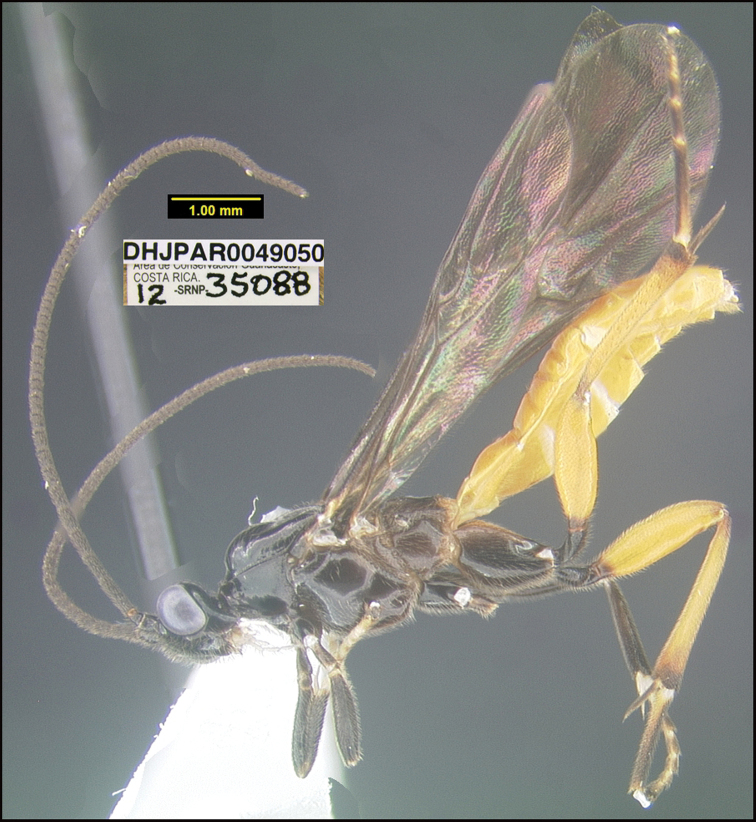
*Lytopylusdavidschindeli*, holotype.

**Holotype** ?: Costa Rica: Guanacaste, Area de Conservación Guanacaste, Sector Cacao, Estación Cacao, 1150 m, 10.92691 -85.46822; host caterpillar collection date: 06/iii/2012, parasitoid eclosion: 02/iv/2012; depository CNC, holotype voucher code: DHJPAR0049050.

##### Holotype host data.

elachJanzen01 Janzen185 (Depressariidae) feeding on *Prunusannularis* (Rosaceae), caterpillar voucher code:12-SRNP-35088.

##### Etymology.

*Lytopylusdavidschindeli* is named in honor of David Schindel of the greater Washington, D.C. area and formerly the US National Science Foundation for his decades of understanding the unconventional traits of the biodiversity inventory of Costa Rica’s Área de Conservación Guanacaste.

### ?Alysiinae

The key by Wharton (1997) is outdated but it is the only available key to Alysiinae genera of the New World. Members of the subfamily are parasitoids of cyclorrhaphous Diptera.

#### ? Gnathopleura josequesadai

Taxon classificationAnimaliaHymenopteraBraconidae

Sharkey sp. nov.

683AB113-1ACF-5427-81F8-95B3CF8F7C11

http://zoobank.org/0D07C3EF-ED94-4933-BD2F-33F027916C6A

##### Diagnostics.

Figure 11.

##### BOLD data.

BIN: BOLD:AAE0055; nearest neighbor: *Gnathopleura* sp. BOLD:AEF6891; distance to nearest neighbor is 7.99%. Consensus barcode GTATTATATTTTATATTTGGTATTTGAGCTGGTATAGTAGGGTTATCTATAAGATTAATTATTCGGTTAGAATTAGGTATACCTGGRTCTTTATTAATAAATGATCAAATTTATAATAGTATAGTAACAGCYCATGCATTTGTCATAATTTTTTTTATAGTTATACCTGTAATAATTGGTGGATTTGGTAATTGATTAGTTCCTTTAATGTTAGGATCTCCTGATATAGCTTTCCCACGAATAAATAATATAAGATTTTGACTTTTAATTCCATCTTTATTGTTATTATTATTAAGAAGAGTATTAAATATTGGTGTAGGAACAGGGTGAACAGTTTATCCACCTTTATCGTCAGGAATTGGTCATAGAGGGATTTCTGTTGATTTAGCTATTTTTTCTTTACATTTGGCTGGKGTATCYTCAATTATAGGGGTTATTAATTTTTTAACTACAATTTTTAATATAAAATCTTGCATGATTAAAATAGATCAGTTAAGGTTATTTATTTGATCTATTTTAATTACAGCTATTTTATTATTATTATCTTTACCTGTTTTAGCAGGTGCAATTACAATATTATTAACTGATCGAAATTTAAATACTACTTTTTTTGATTTTTCAGGTGGTGGGGATCCAATTTTATTTCAACATTTATTT

##### Morphological data.

*Gnathopleurajosequesadai* keys to *G.cariosa* in Wharton (1980) but differs in many ways. For example, the first flagellomere is approximately equal in length to the second flagellomere in *G.cariosa* but much shorter than the second in *G.josequesadai*. This species can be morphologically distinguished from its nearest neighbor by the carina separating the propodeum from the metapleuron. This is rough and complete in *G.josequesadai* (Fig. 11) and smooth and restricted to the posterior 1/3 in the specimen in BIN BOLD:AEF6891.

**Figure 11.**
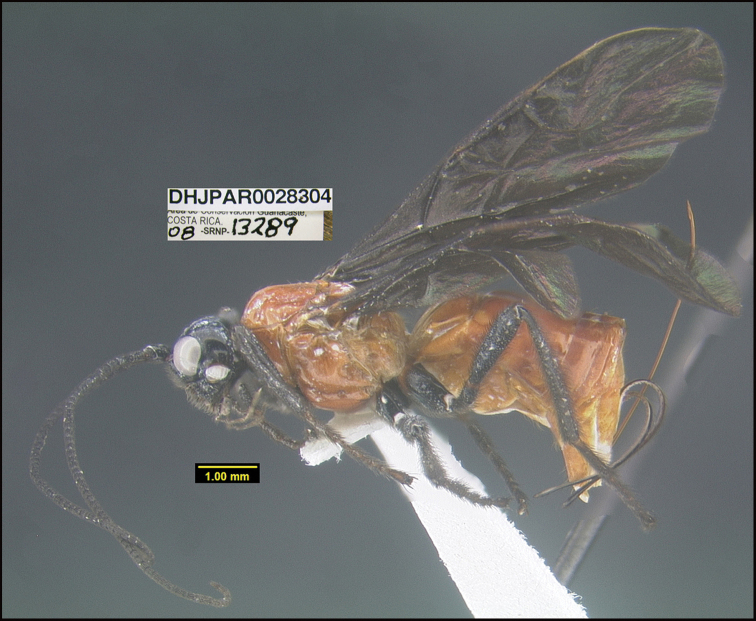
*Gnathopleurajosequesadai*, holotype.

**Holotype** ?: Costa Rica: Guanacaste, Area de Conservación Guanacaste, Sector Santa Rosa, Sendero Natural, 290 m, 10.836 -85.613; host caterpillar collection date: 08/vi/2008, parasitoid eclosion: 11/vii/2008; depository CNC, holotype voucher code: DHJPAR0028304.

##### Holotype host data.

Hyperparasitoid of the fly *Leschenaultia* Wood30DHJ01(Tachinidae) which is a primary parasitoid of *Pachyliaficus* (Sphingidae) feeding on *Macluratinctoria* (Moraceae). Including the holotype, five specimens were reared from the fly puparia parasitizing the caterpillar, voucher code 08-SRNP-13289.The host flies were identified from their surviving sibs, one of which is DHJPAR0027924 of BIN BOLD:ACE9310.

##### Paratype.

Reared from the same caterpillar as the holotype DHJPAR0028038, DHJPAR0028039, DHJPAR0028040, DHJPAR0028041.

##### Etymology.

*Gnathopleurajosequesadai* is named in honor of José Ramón Quesada Mora, the manager of the 2020–21 BioAlfa Malaise traps for the Hacienda Baru Wildlife Refuge, Costa Rica.

##### Note.

This is the first species of *Gnathopleura* confirmed to be a hyperparasitoid.

### ?Braconinae

Braconines are mostly primarily idiobiont parasitoids of Coleoptera and Lepidoptera, but use many other insect orders as well. A key to the Braconinae genera of the New World is in Sharkey et al. (2021a).

#### ? Bracon andreamezae

Taxon classificationAnimaliaHymenopteraBraconidae

Sharkey sp. nov.

9281613C-E428-552C-94D0-B39D9FD09F6C

http://zoobank.org/C8FDFE7C-5289-4EF6-A9F0-BC1C493AEBBD

##### Diagnostics.

Figures 12, 13.

##### BOLD data.

BIN: BOLD:AAJ8891; nearest neighbor: *Braconfranklinpaniaguai*BOLD:ACK6897; distance to nearest neighbor is 5.64%. Consensus barcode: TATATTATATTTTATACTTGGTATTTGATCTGGTATAATTGGTTTATCAATAAGTTTAATTATTCGGTTAGAATTAAGAATACCAGGAAGTTTATTAAGTAATGATCAAATTTATAATAGAATAGTTACAGCACATGCTTTTGTAATAATTTTTTTTATAGTTATACCAGTGATATTAGGAGGGTTTGGTAATTGATTAATCCCTTTAATATTGGGATCTCCTGATATAGCTTTTCCTCGAATAAATAATATAAGATTTTGATTATTAATTCCTTCATTAATTTTATTATTATTAAGAAGAATATTAAATGTAGGAGTGGGTACTGGTTGAACTATTTATCCTCCATTATCTTCTAACTTAGGGCATAGAGGTGTATCTGTTGATTTAGCTATTTTTTCTTTACATTTAGCTGGTATTTCATCTATTATAGGTTCAATTAATTTTATTTCTACAATTTTAAATATACATTTATTAATATTAAAATTAGATCAATTAACTTTATTTATTTGATCAATTTTTATTACAACTATTTTATTATTATTATCTTTACCTGTTTTAGCAGGAGCTATTACTATATTATTAACTGATCGAAATTTAAATACTTCATTTTTTGATTTTTCTGGAGGAGGGGATCCAATTTTATTTCAACATTTATTT

##### Morphological data.

This species can be morphologically distinguished from its nearest neighbor by having the metasoma dorsally entirely yellow and the mesepisternum dorsally black (Fig. 12) compared to the metasoma dorsally black and the mesepisternum entirely yellow-orange in *B.franklinpaniaguai* (Fig. 14).

**Figure 12.**
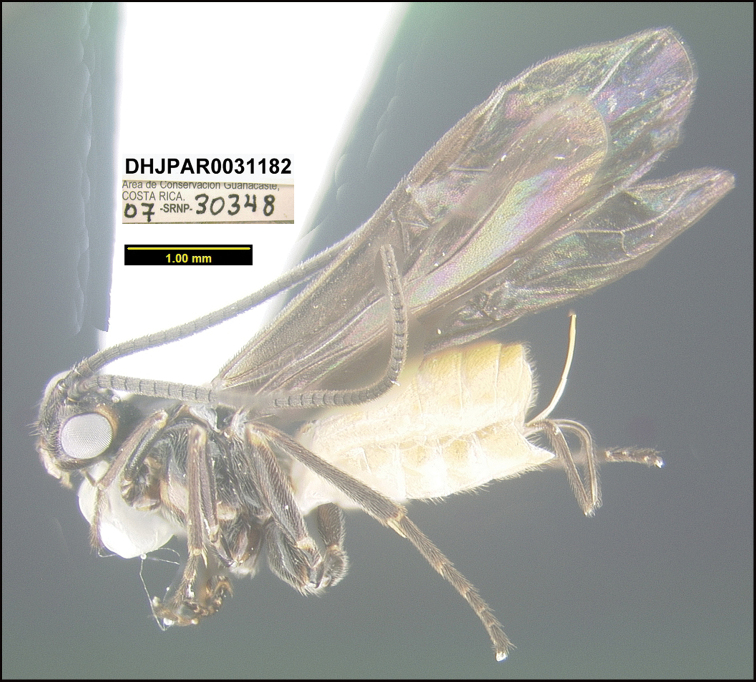
*Braconandreamezae*, holotype.

**Holotype** ?: Costa Rica: Guanacaste, Area de Conservación Guanacaste, Sector Pitilla, Sendero Laguna, 680 m, 10.9888 -85.42336; host caterpillar collection date: 02/i/2007, parasitoid eclosion: 20/i/2007; depository CNC, holotype voucher code: DHJPAR0031182.

##### Holotype host data.

07-SRNP-30348 *Yangunacosyra* (Hesperiidae) feeding on *Chrysochlamysglauca* (Clusiaceae). The species is a gregarious parasitoid; the holotype is one of 56 specimens that emerged from the host, caterpillar voucher code: 07-SRNP-30348.

##### Paratype.

Ten specimens, reared from the same caterpillar as the holotype, were mounted and designated as paratypes (DHJPAR0066400 to DHJPAR0066409), depository CNC.

##### Etymology.

*Braconandreamezae* is named in honor of Ministra Andrea Meza Murillo of the Ministerio de Recursos Naturales y Energía de Costa Rica (MINAE) in recognition of her taking on this difficult ministerial task mid-government.

**Figure 13.**
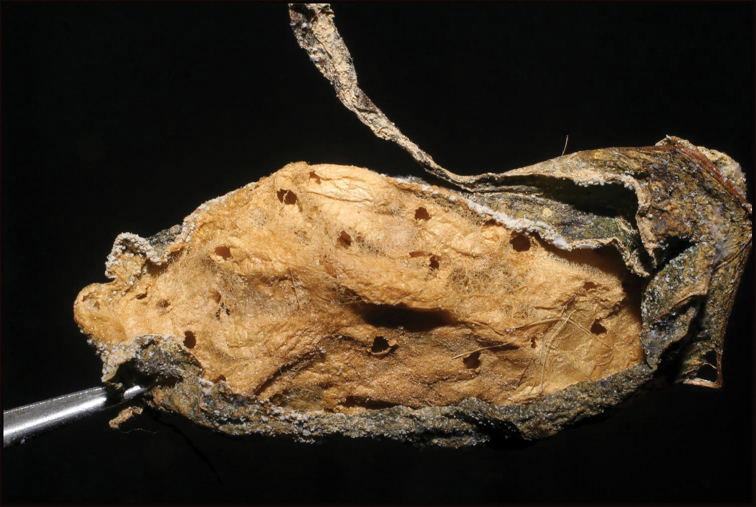
Communal and jointly constructed cocoon of at least 56 sibling larvae of *Braconandreamezae*, one of which is DHJPAR0031182, displaying adult exit holes through the tough silk roof of the same consistency as the floor of the chamber; multiple wasps exited through a single hole. This species of wasp has been reared only twice among 1,391 rearings of solitary *Yangunacosyra* caterpillars for more than 34 years.

#### ? Bracon franklinpaniaguai

Taxon classificationAnimaliaHymenopteraBraconidae

Sharkey sp. nov.

3CC0B67E-7C11-5917-9AE6-2D3E0B4B56D3

http://zoobank.org/45DC951E-E7E0-4C69-9518-E2628FAA99DE

##### Diagnostics.

Figure 14.

##### BOLD data.

BIN: BOLD:ACK6897; nearest neighbor: *Braconalejandromasisi*BOLD:AAA5367; distance to nearest neighbor is 4.49%. Consensus barcode: ATATTATATTTTTTATTTGGAATTTGAGCTGGAATAATTGGTTTATCAATAAGATTAATTATTCGTTTAGAATTAGGTATACCAGGTAGTTTATTAGGTAATGATCAAATTTATAATAGTATAGTTACAGCTCATGCTTTTGTAATAATTTTTTTTATAGTTATACCAGTAATATTAGGAGGATTTGGAAATTGATTAATTCCTTTAATATTAGGAGCTCCTGATATAGCTTTTCCTCGAATAAATAATATAAGATTTTGGTTATTAATTCCTTCATTAATTTTATTATTATTAAGAAGAATATTAAATGTTGGTGTAGGGACAGGTTGAACTATTTATCCTCCTTTATCATCTAGGTTAGGTCATGGAGGTATATCTGTTGATTTAATTATTTTTTCTTTACATTTAGCTGGTATTTCATCTATTATAGGATCTATTAATTTTATTACTACAATTTTAAATATGCATTTATTAATATTAAAGTTAGATCAATTAACTTTATTTATTTGATCAATTTTTATTACAACTATTTTATTATTATTATCTTTACCTGTATTAGCAGGAGCTATTACTATATTATTAACTGATCGAAATTTAAATACTTCATTTTTTGATTTTTCTGGAGGTGGGGATCCAATTTTATTTCAACATTTATTT

##### Morphological data.

This species can be morphologically distinguished from its nearest neighbor by having the mesepisternum entirely orange-yellow, lateral sides of the head orange-yellow, and yellow fore- and mid-tibiae and femora (Fig. 14) compared to the mesepisternum entirely black, head entirely black, and all tibiae and femora black in *B.alejandromasisi* (Sharkey et al. 2021a: fig. 31).

**Figure 14.**
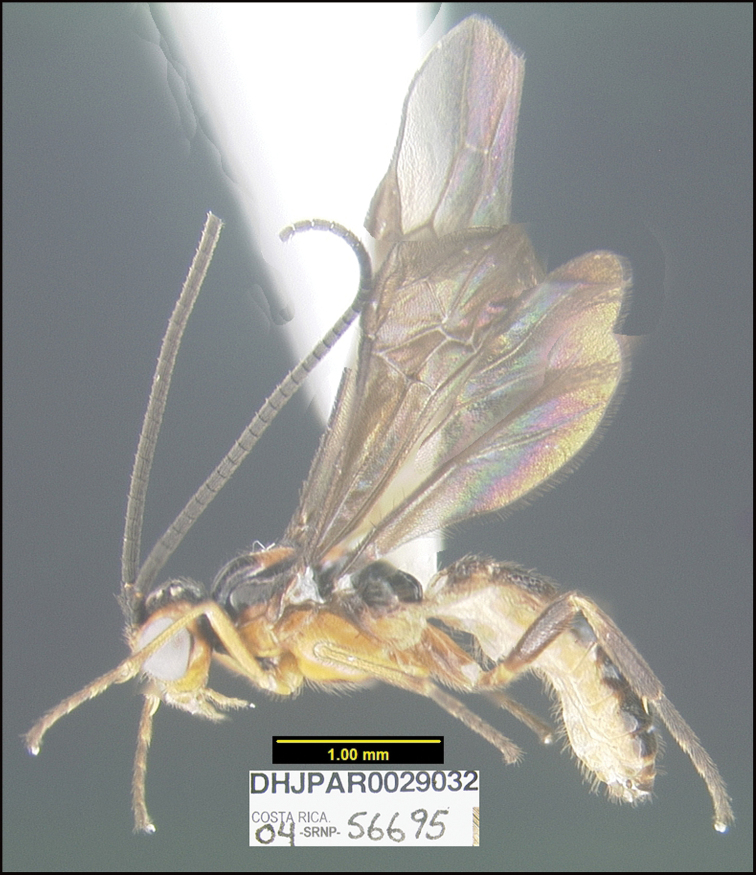
*Braconfranklinpaniaguai*, holotype.

**Holotype** ?: Costa Rica: Guanacaste, Area de Conservación Guanacaste, Sector Pitilla, Pasmompa, 440 m, 11.019 -85.41; host caterpillar collection date: 09/xii/2004, parasitoid eclosion: 28/xii/2004; depository CNC, holotype voucher code: DHJPAR0029032.

##### Holotype host data.

*Fountaineaconfusa* (Nymphalidae) feeding on *Crotonbillbergianus* (Euphorbiaceae), caterpillar voucher code: 04-SRNP-56695.

##### Paratype.

Eight specimens reared from the same host specimen as the holotype were mounted and designated as paratypes (DHJPAR0066410 to DHJPAR0066417), depository CNC.

##### Etymology.

*Braconfranklinpaniaguai* is named in honor of Vice-Minister Franklin Paniagua Alfaro of the Ministerio de Recursos Naturales y Energía de Costa Rica (MINAE) in recognition of his taking on this difficult task mid-government.

#### ? Bracon rafagutierrezi

Taxon classificationAnimaliaHymenopteraBraconidae

Sharkey sp. nov.

4A5F25E2-2180-5096-8BC8-2B5996EE17B3

http://zoobank.org/0093066D-6B8F-4674-9D40-9538CD2ECE5C

##### Diagnostics.

Figure 15.

##### BOLD data.

BIN: BOLD:ACB1290; nearest neighbor: *Bracon* sp.BOLD:AAV0639; distance to nearest neighbor is 2.24%. Consensus barcode: GATTTTATATTTTTTATTTGGTATATGAGCAGGTATAGTTGGTTTATCAATAAGGTTAATTATTCGATTAGAATTAGGTATACCTGGGAGTTTACTAGGTAATGATCAAATTTATAATAGTATAGTGACTGCTCATGCTTTTATTATAATTTTTTTTATAGTTATACCTGTAATATTAGGAGGATTTGGTAATTGATTAATTCCTTTAATATTAGGAGCTCCAGATATAGCTTTTCCTCGTTTAAATAATATAAGATTTTGGTTATTATTTCCTTCTTTAATTTTATTATTATTAAGAAGAATTTTAAATGTTGGTGTAGGTACTGGGTGAACAATATACCCACCTTTGTCATCTAGATTAGGTCATAGAGGGTTATCTGTTGATTTAGCTATTTTTTCTTTACATTTAGCTGGGGTTTCTTCTATTTTAGGTTCAGTTAATTTTATTACAACAATTTTAAATATACATTTATTAATATTAAAATTAGATCAATTAACTTTATTAATTTGATCAATTTTTATTACAACTATTTTATTATTATTATCTTTACCTGTTTTAGCTGGTGCTATTACTATATTATTAACAGATCGAAATTTAAATACTTCTTTTTTTGATTTTTCTGGTGGAGGGGATCCTATTTTATTTCAACATTTATTT

##### Morphological data.

This species can be morphologically distinguished from its nearest neighbor by having all femora dark brown and the metasoma dark brown dorsally starting at the third tergum (Fig. 15) compared to all femora yellow and the metasoma yellow to light brown dorsally.

**Figure 15.**
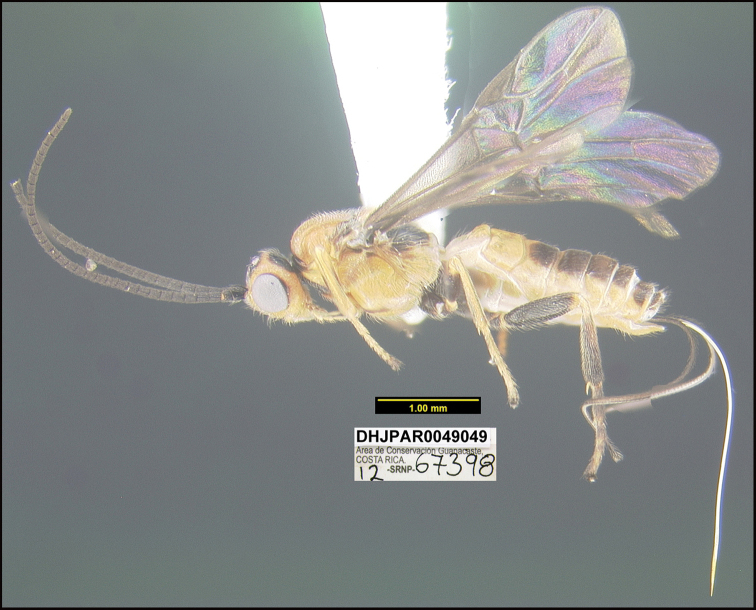
*Braconrafagutierrezi*, holotype.

**Holotype** ?: Costa Rica: Alajuela, Area de Conservación Guanacaste, Sector Rincon Rain Forest, Palomo, 96 m, 10.962 -85.28; host caterpillar collection date: 05/iii/2012, parasitoid eclosion: 18/iii/2012; depository CNC, holotype voucher code: DHJPAR0049049.

##### Holotype host data.

*Cosmorrhynchaalbistrigulana* (Tortricidae) feeding on *Dialiumguianense* (Fabaceae). This is one of the only two species of *Bracon* reared by us that is solitary; the ten species treated by Sharkey et al. (2021a) are all gregarious. It was reared from a very small caterpillar; caterpillar voucher code: 12-SRNP-67398.

##### Etymology.

*Braconrafagutierrezi* is named in honor of SINAC Director Rafa Gutiérrez Rojas of the Ministerio de Recursos Naturales y Energía de Costa Rica (MINAE) in recognition of his taking on this difficult task mid-government.

#### ? Bracon guillermoblancoi

Taxon classificationAnimaliaHymenopteraBraconidae

Sharkey sp. nov.

CE2B1B46-C058-572B-A15B-75CF854B1A96

http://zoobank.org/2E651361-5D12-419B-8A55-A2504505C00E

##### Diagnostics.

Figure 16.

##### BOLD data.

BIN: BOLD:AAT8852; nearest neighbor: *Bracon* sp. BOLD:AED7502; distance to nearest neighbor is 8.17%. Consensus barcode: TATTTTATATTTTTTATTTGGTATATGATCAGGAATAATTGGTTTATCAATAAGTTTAATTATTCGATTAGAATTAGGGATACCTGGAAGATTATTAGGTAATGATCAAATTTATAATAGAATAGTTACAGCTCATGCTTTTATTATAATTTTTTTTATAGTTATACCAATTATATTAGGAGGATTTGGGAATTGATTAATTCCTTTAATATTAGGAGCTCCTGATATAGCTTTTCCTCGTTTAAATAATATAAGATTTTGATTATTAATTCCTTCATTAACTTTATTATTATTAAGAAGAATTTTAAATGTAGGTGTAGGAACTGGGTGAACAATGTATCCTCCATTATCATCTAATTTAGGTCATAGAGGTTTATCTGTAGATTTAGCTATTTTTTCTTTACATTTAGCTGGAATTTCTTCTATTATAGGTTCAATAAATTTTATTACTACTATTTTAAATATACATTTAAAAATATTAAAACTTGATCAATTAACGTTATTAATTTGATCTATTTTTATTACTACAATTTTGTTATTATTATCTTTACCTGTTTTAGCTGGGGCAATTACTATACTATTAACTGATCGAAATTTAAATACTTCATTTTTTGATTTTTCTGGGGGAGGAGACCCAATTTTATTCCAACATTTATTT.

##### Morphological data.

There is only one low-quality image on BOLD for the nearest neighbor, but the p-distance makes it doubtful that it is conspecific.

**Holotype** ?: Costa Rica: Guanacaste, Area de Conservación Guanacaste, Sector Mundo Nuevo, Mamones, 365 m, 10.771 -85.429; host caterpillar collection date: 01/viii/2010, parasitoid eclosion: 12/viii/2010; depository CNC, holotype voucher code: DHJPAR0040470.

##### Holotype host data.

*Dysodiasica* (Thyrididae) feeding on *Pipermarginatum* (Piperaceae). This is one of the only two species of *Bracon* reared by us that is solitary; the ten species treated by Sharkey et al. (2021a) are all gregarious. It was reared from a very small caterpillar; caterpillar voucher code: 10-SRNP-56246.

##### Etymology.

*Braconguillermoblancoi* is named in honor of Guillermo Blanco, the BioAlfa Malaise traps manager for Parque Nacional Isla del Coco, ACMIC (Área de Conservación Marino Isla del Coco), Costa Rica.

**Figure 16.**
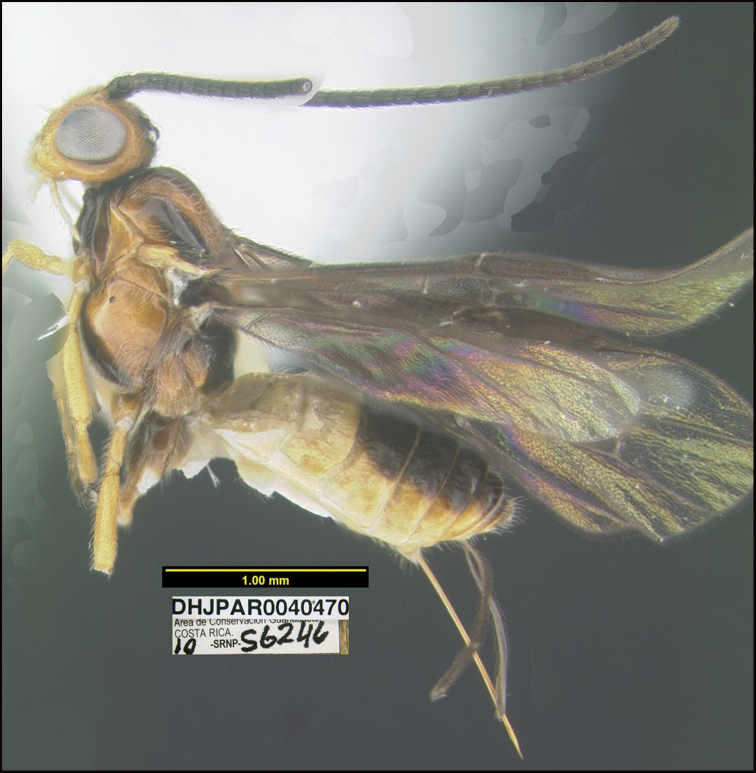
*Braconguillermoblancoi*, holotype.

#### ? Bracon oscarmasisi

Taxon classificationAnimaliaHymenopteraBraconidae

Sharkey sp. nov.

CDA27D08-BB75-5E8B-9802-9C1D254958FE

http://zoobank.org/48249926-80DE-4AB4-98E2-D204D3A3CFB7

##### Diagnostics.

Figures 17, 18.

##### BOLD data.

BIN: BOLD:AAY4686; nearest neighbor: *Bracon* sp. BOLD:AEF4783; distance to nearest neighbor is 6.09%. Consensus barcode: AGTTTTGTATTTTTTATTTGGTATATGAGCTGGTATAGTTGGTTTATCAATAAGTTTAATTATTCGTTTAGAGTTAGGTATACCTGGAAGTTTATTAGGTAATGATCAAATTTATAATAGAATAGTTACAGCTCATGCTTTTGTTATAATTTTTTTTATAGTTATACCTGTTATAATTGGAGGATTTGGTAATTGATTAATTCCTTTAATATTAGGAGCTCCTGATATAGCTTTTCCTCGAATAAATAATATGAGATTTTGGTTATTAGTTCCTTCATTAACTTTATTATTATTAAGTAGAATTTTAAATGTAGGGGTAGGTACAGGTTGGACAATATATCCACCTTTATCTTCAAGTTTAGGTCATAGAGGGTTATCTGTTGATTTAGCTATTTTTTCTTTACATTTAGCTGGTGTTTCTTCAATTATAGGGGCAATAAATTTTATTACTACTATTTTAAATATGCATTTATTAATATTAAAATTAGATCAGTTAACTTTATTAGTTTGATCAATTTTTATTACTACTATTTTATTATTATTATCTTTACCTGTTTTAGCAGGAGCAATTACAATATTATTAACTGATCGAAATTTAAATACTTCTTTTTTTGATTTTTCAGGAGGTGGAGATCCTATTTTATTTCAACATTTATTT.

##### Morphological data.

This species can be morphologically distinguished from its nearest neighbor by having the hind femur dark brown (Fig. 17) compared to yellow.

**Figure 17.**
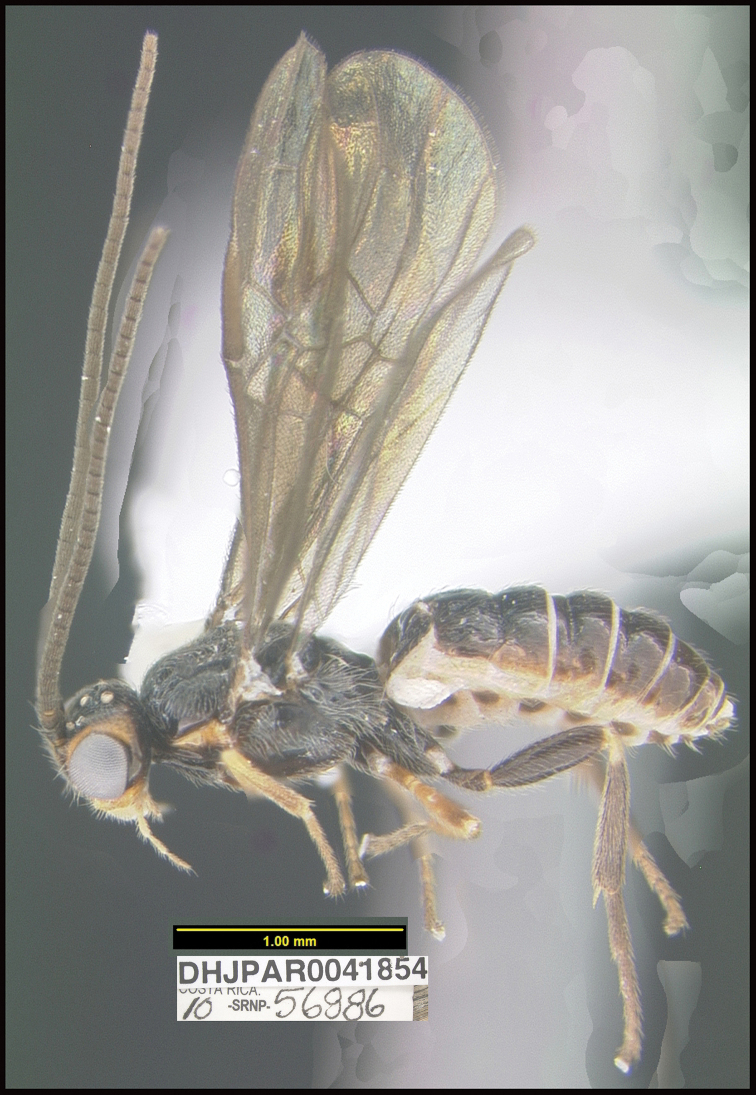
*Braconoscarmasisi*, holotype.

**Holotype** ?: Costa Rica: Guanacaste, Area de Conservación Guanacaste, Sector Mundo Nuevo, Punta Plancha, 420 m, 10.742 -85.427; host caterpillar collection date: 17/x/2010, parasitoid eclosion: 28/x/2010; depository CNC, holotype voucher code: DHJPAR0041854.

##### Holotype host data.

Gregarious parasitoid of *Anadasmus* Janzen25 (Depressariidae) feeding on *Mespilodaphneveraguensis* (Lauraceae); four specimens emerged from the host, caterpillar voucher code: 10-SRNP-56886.

##### Paratype.

Two males, same data as holotype (DHJPAR0066418, DHJPAR0066419) depository CNC.

##### Etymology.

*Braconoscarmasisi* is named in honor of Oscar Masis, the BioAlfa Malaise traps manager for Parque Nacional Los Quetzales, ACOPAC (Área de Conservación Pacífico Central), Costa Rica.

**Figure 18.**
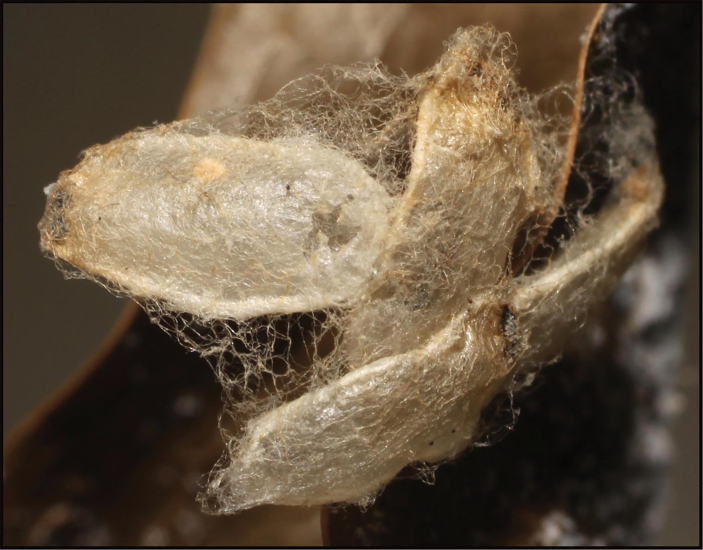
*Braconoscarmasisi*, remains of pupal chamber of host caterpillar, *Anadasmus* Janzen25.

#### ? Bracon pauldimaurai

Taxon classificationAnimaliaHymenopteraBraconidae

Sharkey sp. nov.

6C04CB63-B7C7-59F2-AAC1-E1415E3F7692

http://zoobank.org/723581AA-45CD-4F42-9AB1-CED38EBEA383

##### Diagnostics.

Figure 19.

##### BOLD data.

BIN: BOLD:AEF4305; nearest neighbor: *Bracon* sp. BOLD:ACG3693; distance to nearest neighbor is 9.62%. Consensus barcode: TGTTTTATATTTTTTATTTGGTATATGAGCTGGGATACTAGGTCTATCAATAAGATTAATTATCCGACTAGAGCTCGGAATACCGGGAAGTTTACTTGGTAATGACCAAATTTACAATAGAATAGTAACAGCTCATGCTTTTGTAATAATTTTTTTTATAGTTATACCTGTAATAGTAGGAGGATTTGGAAATTGACTATTACCTTTAATATTAGGAGCCCCTGATATAGCATTTCCTCGTTTAAATAATATAAGATTTTGATTACTTATTCCTTCCCTAACTTTATTATTAATAAGAAGAATTTTAAATGTAGGAGTAGGGACTGGATGAACAGTTTATCCTCCTTTATCCTCTTCACTAGGTCATAGAGGGTTATCAGTTGATTTGGCTATTTTTTCTTTACATATTGCAGGAATTTCCTCAATTTTGGGGGCTATTAACTTTATTTCAACTATTTTAAATATACATTTATTAATTTTAAAACTAGATCAACTAACATTATTAATTTGATCAATTTTTATTACAGCTATTTTATTATTATTATCTTTACCAGTATTAGCAGGAGCTATCACAATATTATTAACCGATCGAAATTTAAATACATCTTTTTTTGATTTTTCAGGAGGGGGTGACCCAATTTTATTTCAACATTTATTT.

##### Morphological data.

This species can be morphologically distinguished from its nearest neighbor by having large portions of the mesoscutum and mesepimeron brown (Fig. 19) compared to entirely black.

**Figure 19.**
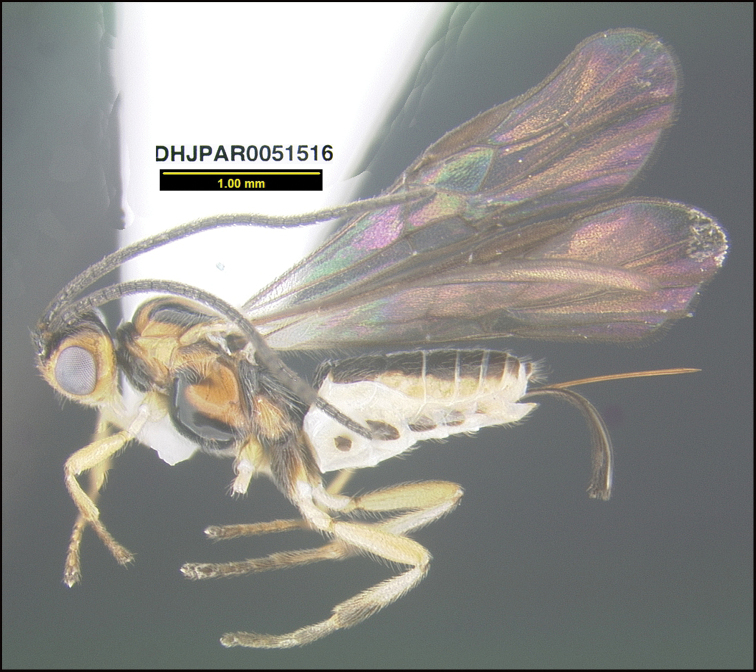
*Braconpauldimaurai*, holotype.

**Holotype** ?: Costa Rica: Guanacaste, Area de Conservación Guanacaste, Sector Cacao, Sendero Cima, 1460 m, 10.933 -85.457, 23/iii/2009, Malaise trap, depository CNC, holotype voucher code: DHJPAR0051516.

##### Etymology.

*Braconpauldimaurai* is named in honor of Paul Dimaura of Boston, Massachusetts for his decades of support of the University of Pennsylvania in general and D. H. Janzen’s position as a professor of conservation biology specifically.

#### ? Bracon shebadimaurae

Taxon classificationAnimaliaHymenopteraBraconidae

Sharkey sp. nov.

D39CF64B-0CA1-5A5C-ABC6-CE4C0016B05C

http://zoobank.org/E87C71B3-2C89-4EB5-AFE9-47FD75A1C533

##### Diagnostics.

Figure 20.

##### BOLD data.

BIN: BOLD:ADW8464; nearest neighbor: *Bracon* sp. BOLD:AEB6448; distance to nearest neighbor is 4.81%. Consensus barcode: TTTATATTTTTTATTTGGTATATGAGCAGGAATATTAGGATTATCACTAAGTTTAATTATTCGTTTAGAATTAGGGATACCTGGAAGATTATTAGGTAATGATCAAATTTATAATAGTATAGTTACAGCTCATGCATTTGTTATAATTTTTTTTATAGTTATACCAGTAATATTAGGAGGATTTGGTAATTGATTATTACCTTTAATATTAGGGGCTCCTGATATAGCTTTCCCACGAATAAATAATATAAGATTTTGGTTAATTATCCCTTCTTTAATTTTATTATTAATAAGAAGAATTTTAAATGTAGGAGTAGGAACAGGTTGAACAGTTTATCCTCCTTTATCATCTTCATTAGGGCATAGTGGGTTATCTGTTGATTTAGCTATTTTTTCTTTACATATTGCAGGAATTTCTTCAATTATAGGTTCAATTAATTTTATTACTACTATTTTTAATATACATTTATTTAAATTAAAATTAGATCAATTAACATTATTAATTTGATCTATTTTTATCACAACTATTTTACTTTTATTATCTTTACCAGTTTTAGCGGGGGGAATTACTATATTATTAACAGATCGTAATTTAAATACATCATTTTTTGATTTTTCTGGAGGGGGAGATCCTGTTTTATTTCAACATTTATT.

##### Morphological data.

No images of the unnamed nearest neighbor are available.

**Holotype** ?: Costa Rica: Guanacaste, Area de Conservación Guanacaste, Sector Pailas Dos, PL12-1, 828 m, 10.7642 -85.335, 14/v/2015, Malaise trap, depository CNC, holotype voucher code: BIOUG44786-F08, GenBank accession MW627534.

##### Etymology.

*Braconshebadimaurae* is named in honor of Sheba Dimaura of Boston, Massachusetts for her decades of support of the University of Pennsylvania in general and D. H. Janzen’s position as a Professor of Conservation Biology.

**Figure 20.**
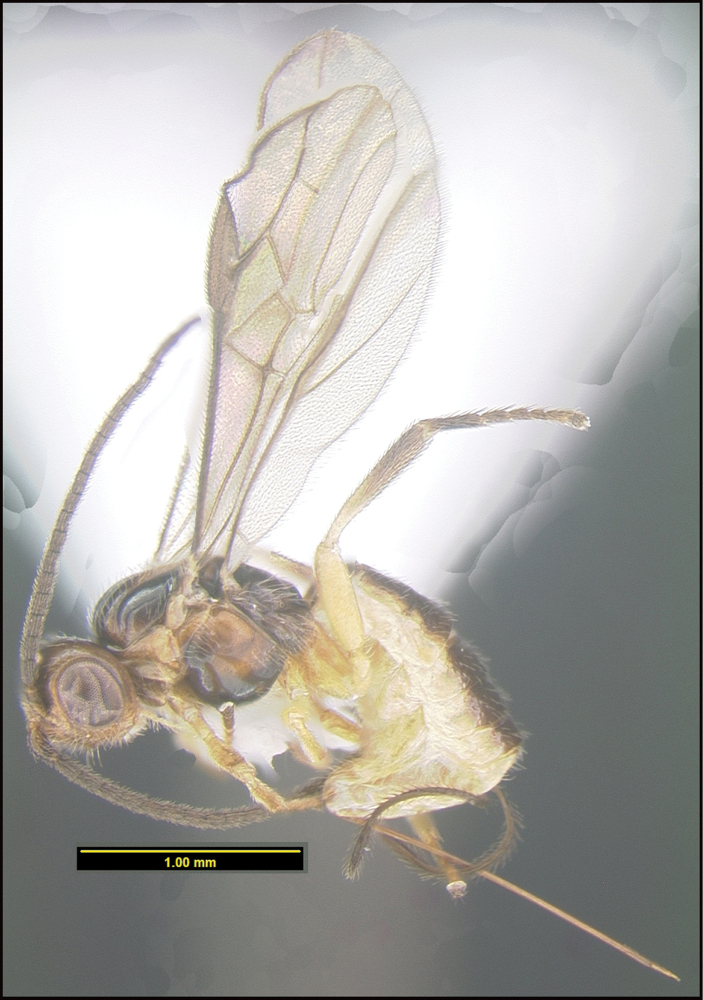
*Braconshebadimaurae*, holotype.

#### ? Sacirema karendimaurae

Taxon classificationAnimaliaHymenopteraBraconidae

Sharkey sp. nov.

1A4A7A9B-09BA-58BA-87AA-4A05BC58362F

http://zoobank.org/5BE4D1E3-9F52-444D-8F49-AC0130EEE9FF

##### Diagnostics.

Figure 21.

##### BOLD data.

BIN: BOLD:ADY0104; nearest neighbor: *Sacirema* sp. BOLD:AEH2057; distance to nearest neighbor is 2.24%. Consensus barcode: TTTATATTTTTTATTTGGGATATGATCTGGTATATTAGGTTTATCAATAAGTTTAATTATTCGATTAGAACTTGGAATACCATCAAGTTTATTAACAAATGATCAAATTTATAATAGAATAGTAACTGCCCATGCATTTGTCATAATTTTTTTTATAGTTATACCAATTATAATTGGTGGATTTGGAAATTGATTAATTCCTTTAATATTAAGAGCTCCAGATATAGCTTTCCCTCGTATAAATAATATAAGTTTTTGATTACTAATTCCTTCTTTAATAATATTAATTTTAAGAAGAATTATTAATACAGGTGTAGGTACTGGTTGAACAGTTTACCCTCCTTTATCTTCTTCTATAGGACATAGAGGAATTTCAGTTGATTTAGCAATTTTTTCTTTACATTTAGCTGGAGCTTCCTCAATTATAGGGTCTATTAATTTTATTTCAACTATTATTAATATACGACTTTATTTAATAAAAATAGATCAATTAACATTATTAATTTGATCTATTTTTATTACTACAATTTTATTATTATTATCATTACCAGTTCTAGCTGGGGCAATCACAATATTATTAACAGATCGAAATTTAAATACTACTTTTTTTGATTTTTCAGGAGGTGGGGATCCAATTTTATTCCAACACTTAT.

This species differs from the three described species of *Sacirema* (Papp 2007) in many ways. The easiest to see is that none of the other three species has a predominantly yellow meso- and metasoma. The generic placement of this species is somewhat uncertain as are those of other specimens in the group of braconine genera with a medial area of the face delimited by longitudinal grooves or ridges.

##### Morphological data.

No images of the unnamed nearest neighbor are available, and when it is described, it should be carefully compared to *Saciremakarendimaurae*.

**Holotype** ?: Costa Rica: Guanacaste, Area de Conservación Guanacaste, Sector Pailas Dos, PL12-3, 820 m, 10.7631 -85.3344, 08/i/2015, Malaise trap, depository CNC, holotype voucher code: BIOUG44686-A07. GenBank accession MW627576.

##### Etymology.

*Saciremakarendimaurae* is named in honor of Karen Dimaura of Boston, Massachusetts for her decades of support of the University of Pennsylvania in general and D. H. Janzen’s position as a professor of conservation biology.

**Figure 21.**
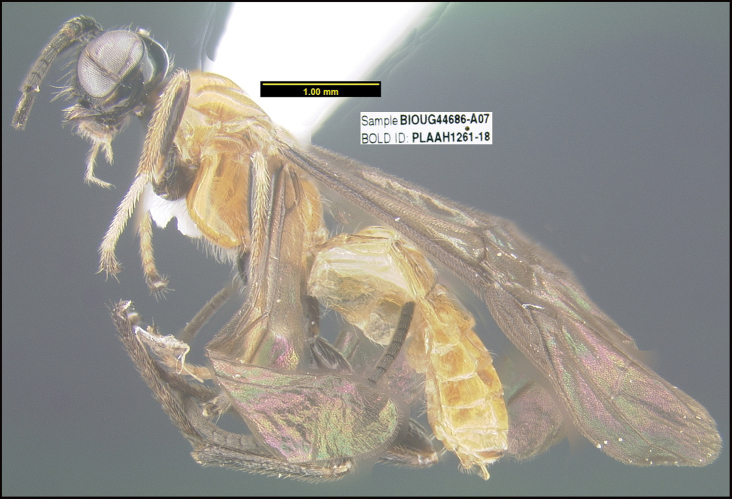
*Saciremakarendimaurae*, holotype.

### ?Cheloninae

Cheloninae are egg-larval parasitoids of Lepidoptera. A key to the genera of the New World is included in Sharkey et al. (2021a).

#### ? Chelonus minorzunigai

Taxon classificationAnimaliaHymenopteraBraconidae

Sharkey sp. nov.

39B3C6C8-0635-5A32-BAE4-387A3705BAD2

http://zoobank.org/6B54FDAF-3228-4F7D-B0B8-8B61B5FDBD6C

##### Diagnostics.

Figure 22.

##### BOLD data.

BIN: BOLD:AEB3509; nearest neighbor: *Chelonusjeffmilleri*BOLD:ACF0845; distance to nearest neighbor is 4.81%. Consensus barcode: AATATTATATTTTGTTTTTGGAATTTGGAGTGGAATAATAGGTTTATCATTAAGATTAATAATTCGTATAGAATTAAGAAGTGTAATAAGATTATTTTATAATGATCAATTATATAATAGAGTTGTAACTATACATGCTTTTATTATAATTTTTTTTATAGTTATACCTTTAATAATTGGAGGATTTGGAAATTGATTAATTCCTTTAATATTAGGATTATCTGATATAATTTTTCCTCGAATAAATAATATAAGATTTTGATTATTAATTCCTTCAATTATTTTATTAATTATAGGAGGGTTTGTTAATATAGGAGCTGGGACAGGATGAACAGTTTATCCTCCATTATCATTATTAATAGGTCATAGAGGAGTTTCAGTAGATTTATCTATTTTTTCTTTACATTTAGCAGGAGTTTCATCTATTATAGGATCAATTAATTTTATTGTTACTATTATAAATACTTGATTACATTATAAATATATAGATAAATACCCATTATTTGTTTGATCAGTTTTTATTACAACTATTTTATTATTATTATCATTACCAGTTTTGGCTGGTGCAATTACTATGTTATTAAGAGATCGAAATTTAAATACAAGATTTTTTGATCCATCAGGAGGAGGAGATCCTGTATTATACCAACATTTGTTT.

##### Morphological data.

This species can be morphologically distinguished from its nearest neighbor by having the hind tibia entirely black (Fig. 22) whereas that of *C.jeffmilleri* has a light brownish yellow patch near the base of the hind tibia, which is otherwise black (Sharkey et al. 2021a: 164, fig. 101).

**Figure 22.**
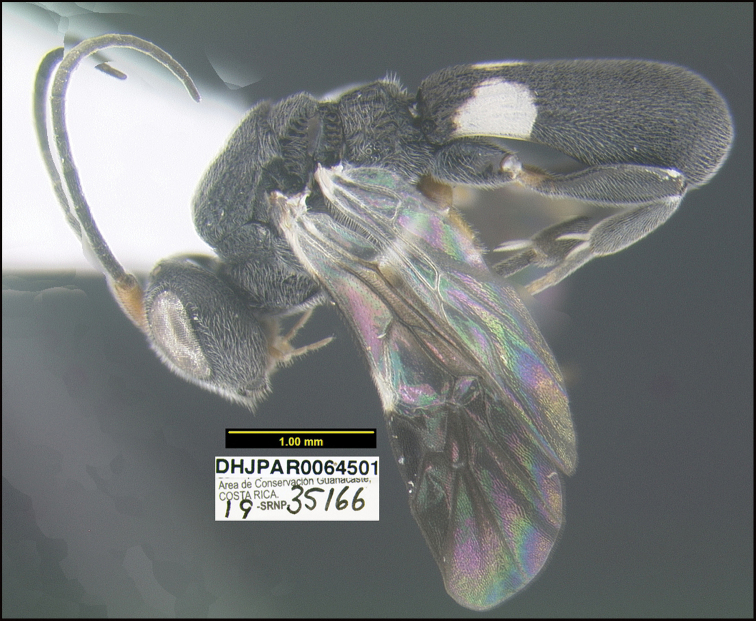
*Chelonusminorzunigai*, holotype.

**Holotype** ?: Costa Rica: Guanacaste, Area de Conservación Guanacaste, Sector Cacao, Estación Cacao, 1150 m, 10.9269 -85.4682; host caterpillar collection date: 07/iii/2019, parasitoid eclosion: 31/iii/2019; depository CNC, holotype voucher code: DHJPAR0064501, GenBank accession: MW627562.

##### Holotype host data.

19-SRNP-35166 *Desmiabenealis* (Crambidae) feeding on *Hameliapatens* (Rubiaceae), caterpillar voucher code:19-SRNP-35166.

##### Paratype.

Same host data as holotype, DHJPAR0064500, DHJPAR0064502, depository CNC.

##### Etymology.

*Chelonusminorzunigai* is named in honor of Minor Zúñiga Siles, the BioAlfa Malaise traps manager for Estación Esquinas, Parque Nacional Tortuguero, ACTO (Área de Conservación Tortuguero), Costa Rica.

### ?Homolobinae

Members of Homolobinae are endoparasitoids of lepidopteran larvae. A key to the genera of the New World is included in Sharkey et al. (2021a).

#### ? Homolobus stevestroudi

Taxon classificationAnimaliaHymenopteraBraconidae

Sharkey sp. nov.

4D94064C-D823-5FDA-B2A0-49F2323CD7DF

http://zoobank.org/D21DFA7D-C461-4F83-9521-79A1E9982771

##### Diagnostics.

Figures 23, 24.

##### BOLD data.

BIN: BOLD:AAA7060. The nearest neighbor: *Homolobus* sp. BOLD:ACM2462 is separated by a p-distance of only 1.12%. Consensus barcode: TATTTTATATTTTATATTTGGAATTTGAGCTGGAATTTTAGGTATATCAATAAGAATTATTATTCGAATAGAATTAAGAATACCAGGTAATTTAATTGGTAACGATCAAATTTATAATAGTATTGTTACTGCTCATGCATTTATTATAATTTTTTTTATAGTTATACCAATTATAATTGGAGGGTTTGGAAATTGATTAATTCCTTTAATATTAGGATGTGTTGATATAGCTTTTCCTCGAATAAATAATATAAGATTTTGATTATTAATTCCATCATTAATTTTATTAATTTTAAGAAGAATTTTAAATGTTGGTGTTGGTACTGGATGAACTGTTTATCCTCCTTTATCTTTAAATATTGGTCATGGAGGTTTATCTGTTGATTTAGCTATTTTTTCTTTACATTTAGCTGGAATTTCTTCAATTATAGGAGCTATTAATTTTATTACTACTATTTTAAATATACGATCTAATTTAATTACAATAGATAAAATTTCTTTATTAAGTTGATCAATTTTAATTACTGTAATTTTATTATTATTATCTTTACCAGTTTTAGCTGGGGCTATTACAATATTATTAACTGATCGTAATTTAAATACATCTTTTTTTGATCCATCTGGTGGAGGGGATCCAATTTTATATCAACATTTATTT.

##### Morphological data.

The specimen keys to *Homolobusinfumator* in van Achterberg’s (1979) key and is very similar morphologically. Subtle differences include the shape of vein R1a of the hind wing, which is longer in *H.stevestroudi* and the size of the basal tooth of the hind tarsal claws, which are longer in *H.stevestroudi*. The convincing difference can be found by looking at the NJ tree produced from the BOLD website; *H.stevestroudi* is found in its own BIN, far removed from any other species of *Homolobus* and particularly distant from specimens identified as *H.infumator* from Norway. The type locality of *H.infumator* is England. All nine specimens in the unnamed nearest neighbor are from Canada. They might represent the same species as the Costa Rican specimens, but more sampling will need to be done between Canada and Costa Rica to confirm or refute. There are no obvious morphological differences based on the BOLD images of the Canadian specimens.

**Figure 23.**
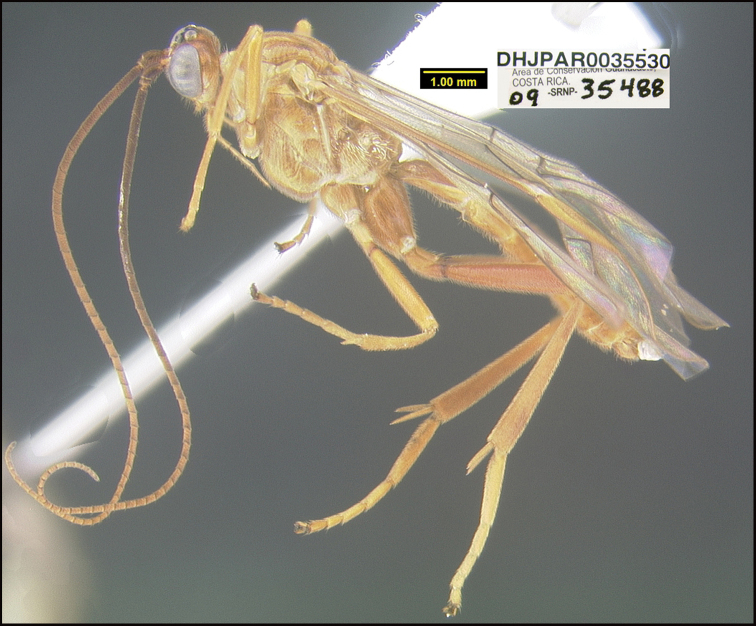
*Homolobusstevestroudi*, holotype.

**Figure 24.**
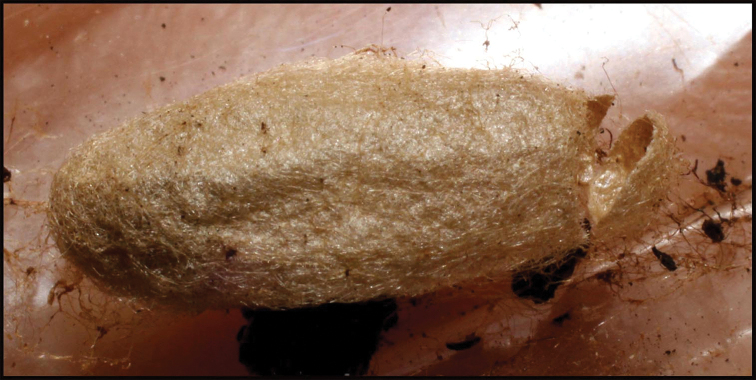
Tough-walled silk cocoon of *Homolobusstevestroudi*. Note shiny surface of the inside visible just inside the cut off right-hand end. That hard smooth surface makes the cocoon wall extremely tough and difficult to penetrate with an insect pin. The terminal circular cut exit hole is characteristic of most genera of large bodied ACG Braconidae and many small ones as well.

**Holotype** ?: Costa Rica: Guanacaste, Area de Conservación Guanacaste, Sector Cacao, Sendero Toma Agua, 1140 m, 10.928 -85.467; host caterpillar collection date: 23/iv/2009, parasitoid eclosion: 18/v/2009; depository CNC, holotype voucher code: DHJPAR0035530, GenBank accession: MW627552.

##### Holotype host data.

*Pherotesiaminuisca* (Geometridae) feeding on *Zygiapalmana* (Fabaceae), caterpillar voucher code: 09-SRNP-35488.

##### Etymology.

*Homolobusstevestroudi* is named in honor of Steve Stroud as the primary supporter of the BioAlfa Malaise trapping at the Hacienda Barú Wildlife Refuge, Savegre, ACOPAC, Costa Rica, as well as decades of support for the Area de Conservación Guanacaste.

### ?Macrocentrinae

Members of all genera are koinobiont endoparasitoids of caterpillars from a wide range of families. Most are solitary, but several gregarious species are known. A key to the genera of the New World is in Sharkey et al. (2021a).

#### ? Macrocentrus michaelstroudi

Taxon classificationAnimaliaHymenopteraBraconidae

Sharkey sp. nov.

C02F5F5C-22FE-545C-8AC9-DDF6868514D7

http://zoobank.org/6E04139A-E5D6-4F29-BC63-DBD88D4EBADA

##### Diagnostics.

Figure 25.

##### BOLD data.

This is the eighth species to be described in BIN BOLD:ACK7466. Consensus barcode: TGTTTTATATTTTTTATTAGGTATTTGATGTGGATTAGTAGGTTTATCTTTAAGGTTACTTATTCGGTTAGAGTTGAGAAATTTAGGAAGATTATTAGGTAATGATCAAATTTATAATGTAGTTGTTACTATACATGCTTTTATTATAATTTTTTTTATGGTGATACCTATTATAATTGGAGGTTTTGGAAATTGATTAATTCCCTTAATATTAGGAGCTCCTGATATAGCTTTCCCTCGTATAAATAATATAAGATTTTGATTATTAATTCCTTCTTTGTTATTAATATTAATAAGAGGATTTATTATAGTTGGTAGTGGAACTGGGTGAACTATATATCCTCCTTTAAGTTCTTTAATTGGGCATAGAAGGTTTTCTGTTGATATAGTAATTTTTTCTTTACATTTAGCAGGGGTTTCTTCAATTATAGGAGCTATTAATTTTATTACAACAATTTTTAATATAAAATTAATTT---TAAAATTAGATCAGATTATATTATTTGTATGATCTGTATTAATTACTGCTTTTTTATTATTACTTTCTTTACCTGTTTTGGCAGGAGGAATTACTATATTATTAACAGATCGTAATTTAAATACTTCTTTTTTTGATTTTTCAGGAGGGGGAGATCCTGTTTTATTTCAACACTTATTT.

##### Morphological data.

In the morphological key to the species in this BIN (Sharkey et al. 2021a), *Macrocentrusmichaelstroudi* will key to *M.gustavogutierrezi*. *Macrocentrusmichaelstroudi* differs in its pale basal flagellomeres, contrasting with the melanic basal flagellomeres of *M.gustavogutierrezi*. The host caterpillar also differs from those of *M.gustavogutierrezi*. It belongs to the group of species with vein M+Cu of the forewing distinctly widened apically.

**Figure 25.**
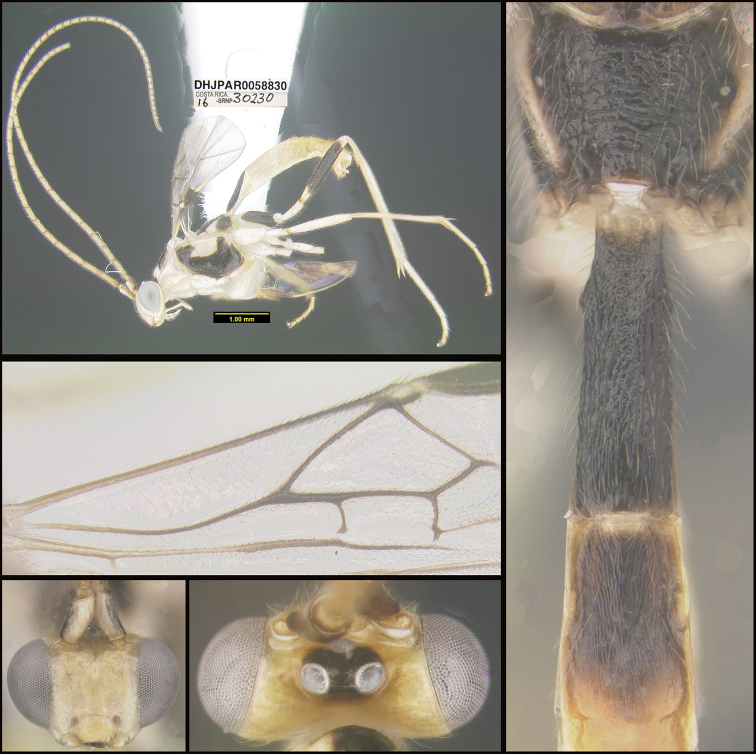
*Macrocentrusmichaelstroudi*, holotype.

**Holotype** ?: Costa Rica: Guanacaste, Area de Conservación Guanacaste, Sector Pitilla, Sendero Rotulo, 510 m, 11.0135 -85.4241; host caterpillar collection date: 22/i/2016, parasitoid eclosion: 01/iii/2016; depository CNC, holotype voucher code: DHJPAR0058830, GenBank accession: MW627584.

##### Holotype host data.

*Phaedropsis* leialisDHJ03 (Crambidae) feeding on *Gouanialupuloides* (Rhamnaceae), caterpillar voucher code: 16-SRNP-30230.

##### Etymology.

*Macrocentrusmichaelstroudi* is named in honor of Michael Stroud Bonilla as the primary supporter of the BioAlfa Malaise trapping at the Hacienda Baru Wildlife Refuge, Savegre, ACOPAC, Costa Rica, as well as decades of support for the Area de Conservación Guanacaste.

### ?Orgilinae

Members of all genera are koinobiont endoparasitoids of caterpillars. A key to the genera of the New World can be found in Sharkey et al. (2021a).

#### ? Stantonia gilbertfuentesi

Taxon classificationAnimaliaHymenopteraBraconidae

Sharkey sp. nov.

AC50A86E-2624-5E92-9CCD-172E1C60F06B

http://zoobank.org/6058EB69-E85F-4CFB-8771-92DD917FA191

##### Diagnostics.

Figure 26.

##### BOLD data.

BIN: BOLD:AEC3983; nearest neighbor: *Stantoniamiriamzunzae*BOLD:ACB1896; distance to nearest neighbor is 4.47%. Consensus barcode: TATATTGTATTTTTTGTTTGGTATATGGTCTGGGGTGTTAGGTTTATCACTAAGTTTAATTATTCGTATAGAATTAGGTCAAATTGGTTCATTTATTGGAAATGATCAAATTTATAATAGTATTGTTACTTCTCATGCTTTTATTATAATTTTTTTTATAGTTATGCCTATTATAATTGGGGGGTTTGGAAATTGATTGATTCCTTTAATATTAGGAAGTGTTGATATAGCTTTCCCTCGAATAAATAATATAAGATTTTGATTATTAATTCCTTCTTTAATATTATTAATTTTAAGAGGATTTATAAATATTGGTGTAGGTACAGGATGAACAGTTTACCCTCCTTTATCATTAAATGTTAGTCATATAGGAATTTCTGTAGATATAGCTATTTTTTCATTACATTTGGCTGGTATTTCTTCAATTATAGGTGCTATTAATTTTATTGTTACTATTATAAATATACGAAATTATGGGGTATTAATAGATAAAATTAGATTATTATCATGATCAATTTTAATTACAGCTATTTTATTATTGTTATCTTTACCTGTGTTAGCTGGTGCTATTACAATATTGTTAACTGACCGTAATTTAAATACATCCTTTTTTGATCCTGCTGGAGGAGGGGATCCTATTTTATATCAACATTTATTT

##### Morphological data.

This species can be morphologically distinguished from its nearest neighbor by having the first metasomal tergite uniformly pale yellowish-orangeand the mesoscutum uniformly yellowish-orange (Fig. 26), contrasting with the first metasomal tergite darkening apically and melanic patches on each of the three mesoscutal lobes in *S.miriamzunzae* (Sharkey et al. 2021a: 502, fig. 354).

**Figure 26.**
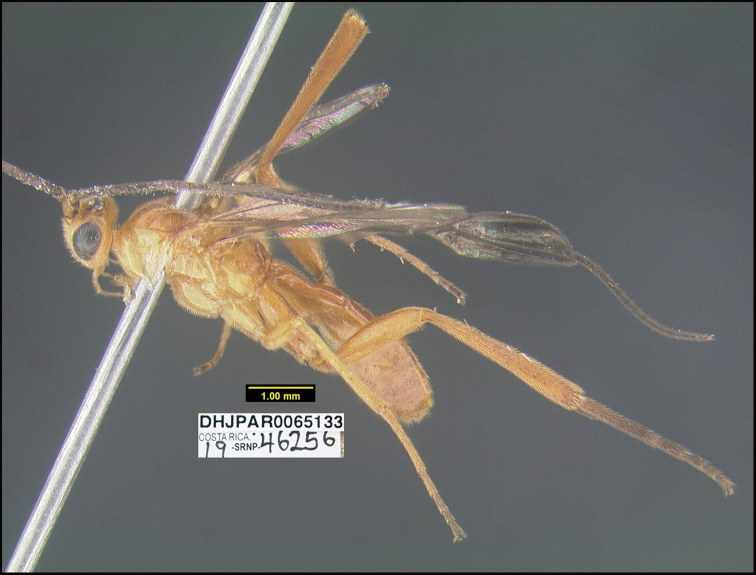
*Stantoniagilbertfuentesi*, holotype.

**Holotype** ?: Costa Rica: Guanacaste, Area de Conservación Guanacaste, Sector Rincon Rain Forest, 369 m, 10.969 -85.32; host caterpillar collection date: 07/ix/2019, parasitoid eclosion: 28/ix/2019; depository CNC, holotype voucher code: DHJPAR0065133, GenBank accession: MW627539.

##### Holotype host data.

*Casandria* Poole01 (Erebidae) feeding on *Vismiabaccifera* (Hypericaceae), caterpillar voucher code:19-SRNP-46256. Erebidae is a new host-family record for *Stantonia*.

##### Etymology.

*Stantoniagilbertfuentesi* is named in honor of Gilbert Fuentes of the Organización de Estudios Tropicales of Costa Rica in recognition of his decades of intensive management of the OET library of tropical publications.

### ?Rhysipolinae

Members of the subfamily are thought to be solitary, koinobiont ectoparasitoids of caterpillars. A diagnosis for the subfamily is included in Sharkey et al. (2021a).

#### ? Rhysipolis stevearonsoni

Taxon classificationAnimaliaHymenopteraBraconidae

Sharkey sp. nov.

B58E733C-3E93-5BB7-918B-A9768C49D776

http://zoobank.org/B5D5C71B-6810-4120-976F-D30A453FF262

##### Diagnostics.

Figure 27.

##### BOLD data.

BIN: BOLD:ADA0151; nearest neighbor: *Rhysipolis* sp. BOLD:ADL9389; distance to nearest neighbor is 2.91%. Consensus barcode: TGTATTATATTTTTTATTTGGAATTTGATCTGGAATAGTAGGTTTGTCTATGAGTTTAATTATTCGTTTAGAGTTAGGTATACCCGGTAGTTTGTTATTTAATGATCAGATTTATAATACGATAGTTACAGCTCATGCTTTTATTATAATTTTTTTTATAGTGATACCTGTAATGATTGGGGGGTTTGGTAATTGGTTAGTTCCATTAATGTTGGGGGCTCCTGATATAGCTTTTCCTCGTATGAATAATATAAGATTTTGATTATTAATTCCTTCTTTAATTTTATTATTTTTGAGGGGATTAGTAAATGTTGGGGTAGGTACTGGATGAACAGTTTATCCTCCTTTATCTTCTTCTATAGGTCATAGAGGTATTTCTGTTGATTTGGCTATTTTTTCTTTACATTTAGCTGGTATTTCTTCTATTATAGGAGCTATTAATTTTATTTCAACAATTTTTAATATGTGTTTATATTCAATTAATATAGATCAAATTAGTTTATTTATTTGATCTATTTTGATTACTGCTTTTTTATTATTGTTGTCTTTACCTGTTTTGGCAGGGGCTATTACTATATTGTTAACGGATCGAAATTTAAATACTTCATTTTTTGATTTTTCTGG

##### Morphological data.

This species can be morphologically distinguished from its nearest neighbor by having its mesoscutum entirely black (Fig. 27) compared to partially orange-brown.

**Figure 27.**
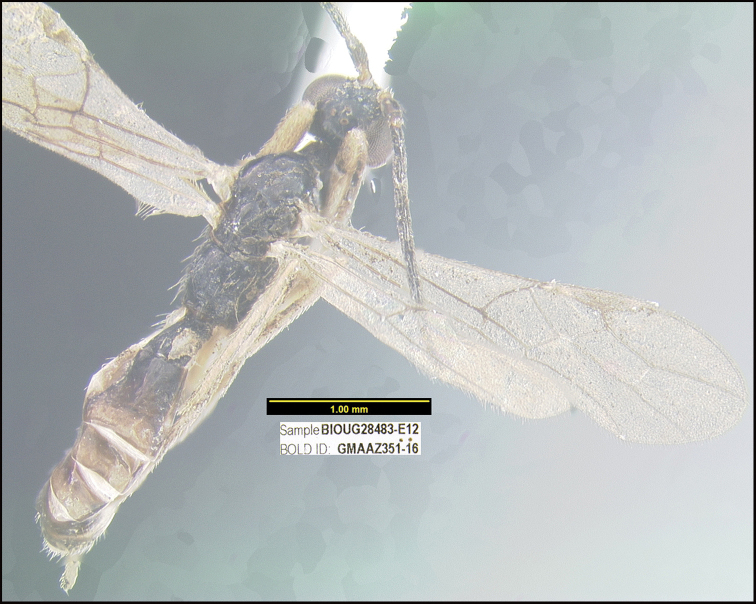
*Rhysipolisstevearonsoni*, holotype.

**Holotype** ?: Costa Rica: Guanacaste, Area de Conservación Guanacaste, Sector San Cristobal, Estación San Gerardo, 575 m, 10.8801 -85.389, 04/viii/2014, Malaise trap, depository CNC, holotype voucher code: BIOUG28483-E12, GenBank accession MW627555.

##### Paratype.

BIOUG27682-G07.

##### Etymology.

*Rhysipolisstevearonsoni* is named in honor of Steve Aronson of San Jose, Costa Rica, in recognition of decades of concern and involvement with the betterment of Costa Rica’s positive relationship with its wild environment, and specifically with providing broadband internet to Área de Conservación Guanacaste as the first Costa Rican Área de Conservación to be so facilitated.

### ?Rogadinae

Members of all genera are koinobiont endoparasitoids of caterpillars from a wide range of families. A key to the genera of the New World is in Sharkey (2021a).

#### ? Aleiodes kaydodgeae

Taxon classificationAnimaliaHymenopteraBraconidae

Sharkey sp. nov.

41DDCF67-4214-5C16-A53A-95B1E9F9DDE6

http://zoobank.org/B8C158F4-07CA-4776-91B7-D7507870695F

##### Diagnostics.

Figure 28.

##### BOLD data.

BIN: BOLD:AEB2985; nearest neighbor: *Aleiodes* sp. BOLD:AAV6245; distance to nearest neighbor is 10.74%. Consensus barcode: AGTATTGTATTTTTTATTTGGTATATGAGCTGGAATAGTTGGTTTATCTATAAGATTAATTATTCGGTTAGAATTAAGAGTTGGGGGGAGAGTCTTAAAAAATGATCAGATTTATAAYGGTATAGTAACTTTGCATGCTTTTGTAATAATTTTTTTTATAGTTATACCTATTATATTAGGAGGGTTTGGAAATTGGTTAGTTCCTTTAATATTGAGAGCTCCAGATATAGCTTTCCCTCGAATAAATAATATAAGATTTTGATTATTAATTCCATCTTTTTTTTTATTATTGATTAGAGGTGTTATTAATTCAGGRGTAGGAACAGGTTGAACAATATATCCTCCTCTTTCTTTATTAATTGGTCATGATGGAATTTCTGTAGATATATCAATTTTTTCTTTACATTTAGCAGGAGCTTCTTCCATTATAGGTTCAATTAATTTTATTTCTACTATTTTTAATATAAAATTAAAAGATTTAAAATTAGATCAAGTTTCTTTATTTGTTTGATCTATTTTAATTACAACAATTTTATTATTGTTATCTTTACCTGTTTTAGCGGGGGCAATTACTATATTATTGACTGATCGAAACTTAAATACAAGATTTTTTGATTTTGCTGGAGGAGGGGATCCAATTTTATTTCAACATTTGTTT.

##### Morphological data.

This species can be morphologically distinguished from its nearest neighbor by having the base of the stigma brown (Fig. 28), contrasting with yellow in the nearest neighbor.

**Figure 28.**
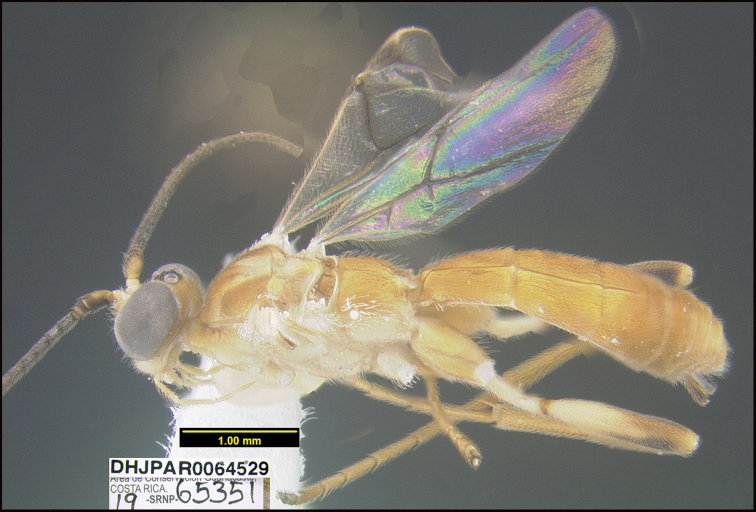
*Aleiodeskaydodgeae*, holotype.

**Holotype** ?: Costa Rica: Guanacaste, Area de Conservación Guanacaste, Sector Brasilia, Gallinazo, 360 m, 11.0183 -85.372; host caterpillar collection date: 10/vii/2019, parasitoid eclosion: 23/vii/2019; depository CNC, holotype voucher code: DHJPAR0064529, GenBank accession: MW627545.

##### Holotype host data.

*Isogona* Poole07 (Erebidae) feeding on *Celtisiguanaea* (Ulmaceae), caterpillar voucher code:19-SRNP-65351.

##### Paratype.

DHJPAR0065341.

##### Etymology.

*Aleiodeskaydodgeae* is named in honor of Kay Dodge of Costa Rica’s Nicoya Peninsula today, original and decades long facilitator of ACG support from Peter Wege (RIP) of the Wege Foundation of Grand Rapids, Michigan, USA.

#### ? Aleiodes kerrydresslerae

Taxon classificationAnimaliaHymenopteraBraconidae

Sharkey sp. nov.

3A4BC25A-6E30-5F9F-A290-6CBA3F6752E7

http://zoobank.org/273165E1-C8BD-43E8-8EED-75004A28DD99

##### Diagnostics.

Figure 29.

##### BOLD data.

BIN: BOLD:AEF3944; nearest neighbor: *Aleiodes* sp. BOLD:AAG1309; distance to nearest neighbor is 8.29%. Consensus barcode: AGTATTATATTTTTTATTTGGAATATGAGCAGGAATAATTGGGATATCAATAAGTTTAATAATCCGATTAGAATTAAGAACAAATGGAAGAATCTTAAAAAATGATCAAATTTATAATGGTATGGTAACTTTACATGCCTTTATTATAATTTTTTTTATAGTAATACCAATTATAATTGGAGGATTTGGAAATTGATTAATTCCTTTAATATTAGGAGCTCCTGACATAGCTTTCCCACGTATAAATAATATAAGATTTTGATTACTAATACCTTCTTTAATACTTTTATTACTTAGAGGAATAATTAATACCGGGGTAGGAACAGGATGAACTATATATCCCCCTTTATCATCACTAATTGGACATAATGGAATTTCAGTAGATATATCTATTTTTTCTTTACACCTTGCAGGGGCTTCTTCAATTATAGGAGCAATCAACTTCATTTCAACTATTTTTAATATAAATATTATAACAATTAAAATAGATCAAATTATACTATTAATTTGATCTATTTTAATTACTACAATCCTTTTATTATTATCTTTACCAGTATTAGCAGGAGCAATTACTATATTACTAACAGATCGAAATTTAAATACAAGATTTTTTGACTTTTCAGGGGGAGGAGACCCTATTTTATTCCAACATCTTTTT.

##### Morphological data.

This species can be morphologically distinguished from its nearest neighbor by its pale stigma (Fig. 29), which is mostly melanic in the nearest neighbor.

**Figure 29.**
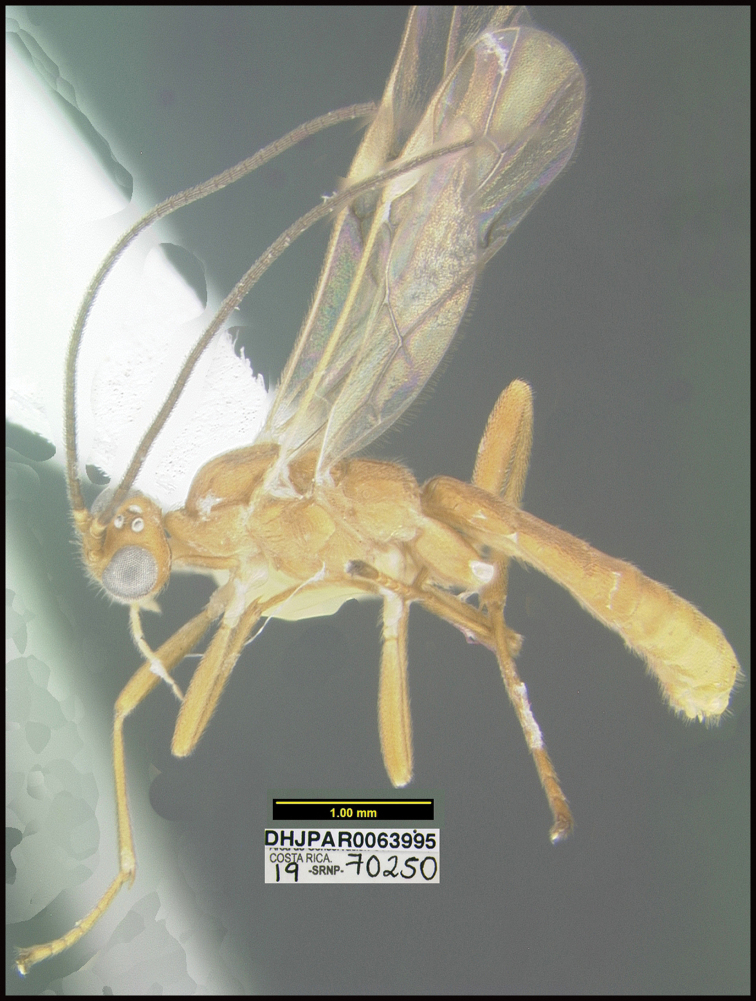
*Aleiodeskerrydresslerae*, holotype.

**Holotype** ?: Costa Rica: Guanacaste, Area de Conservación Guanacaste, Sector Pitilla, Bullas, 440 m, 10.98670 -85.38503; host caterpillar collection date: 25/i/2019, parasitoid eclosion: 13/ii/2019; depository CNC, holotype voucher code: DHJPAR0063995, GenBank accession: MW627548.

##### Holotype host data.

*Anomisgentilis* (Erebidae) feeding on *Peltaeaovata* (Malvaceae), caterpillar voucher code:19-SRNP-70250.

##### Paratype.

DHJPAR0063970.

##### Etymology.

*Aleiodeskerrydresslerae* is named in honor of Kerry Dressler for her life career of deep and intense work and interest in the taxonomy of orchids, and of supporting Bob Dressler’s enthusiasm for the same.

#### ? Aleiodes josesolanoi

Taxon classificationAnimaliaHymenopteraBraconidae

Sharkey sp. nov.

7B3CF334-BA1C-592A-952E-AC5502B0E972

http://zoobank.org/2A3DC697-674E-4EB5-AE11-A15680375504

##### Diagnostics.

Figure 30.

##### BOLD data.

BIN: BOLD:AEB1913; nearest neighbor: *Aleiodes* sp. BOLD:AAM5681; distance to nearest neighbor is 2.24%. Consensus barcode: AATTTTATATTTTTTATTTGGTTTATGAGCAGGAATAATTGGGATATCAATAAGATTAATTATTCGATTAGAATTAAGAACTAGAGGTAGAATTTTAAAAAATGATCAAATTTATAACGGCATAGTAACTTTACATGCATTTATTATAATTTTTTTTATAGTAATACCCATTATAATTGGAGGGTTTGGAAATTGATTAATYCCTCTAATATTAGGAGCCCCTGATATAGCATTTCCTCGAATAAATAATATAAGATTTTGATTACTAATTCCATCATTAATATTTTTATTAATTAGAGGAATTATTAATACAGGTGTAGGAACAGGATGAACAATATATCCTCCATTATCTTCATTAATTGGACATAATAGAATTTCAGTTGATATATCAATTTTTTCTTTACATATAGCAGGTGCTTCATCAATTATAGGAGCTATTAATTTTATTTCAACAATTTTCAATATAAACTTAATAAAAATCAAAATAGAYCAAATTATATTATTAGTTTGATCAGTTTTAATTACAGCCATTTTATTATTACTTTCATTACCTGTTTTAGCAGGAGCAATCACTATATTRTTAACCGACCGTAACTTAAATACAAGTTTTTTTGATTTTTCAGGAGGAGGAGATCCTATTTTATTCCAACATTTATTT.

##### Morphological data.

The nearest neighbor is a sole specimen from Canada. There is no image available on BOLD but due to the distribution, conspecificity is doubtful.

**Holotype** ?: Costa Rica: Guanacaste, Area de Conservación Guanacaste, Sector Del Oro, Meteorologico, 590 m, 11.002 -85.4617; host caterpillar collection date: 25/vi/2019, parasitoid eclosion: 15/vii/2019; depository CNC, holotype voucher code: DHJPAR0064517, GenBank accession: MW627567.

##### Holotype host data.

*Herbitamedama* (Geometridae) feeding on *Dendropanaxarboreus* (Araliaceae), caterpillar voucher code:19-SRNP-20457.

**Figure 30.**
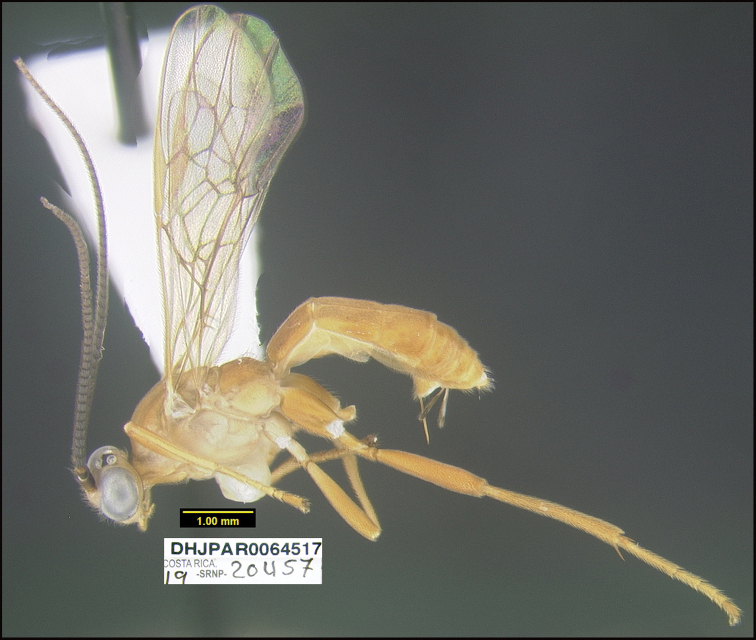
*Aleiodesjosesolanoi*, holotype.

##### Paratype.

DHJPAR0064001 (DHJPAR0062033 not barcoded), host data *Prenestascyllalis* feeding on *Forsteroniaspicata* (Apocynaceae), depository CNC.

##### Etymology.

*Aleiodesjosesolanoi* is named in honor of Jose Andrés Solano, the BioAlfa Malaise traps manager for Estación El Ceibo, Parque Nacional Braulio Carrillo, ACC, Costa Rica.

#### ? Aleiodes juniorporrasi

Taxon classificationAnimaliaHymenopteraBraconidae

Sharkey sp. nov.

7F53D704-D338-583F-AF41-E21CF4F5BA18

http://zoobank.org/474FE466-BEF5-4451-8623-252D716F6B6F

##### Diagnostics.

Figure 31.

##### BOLD data.

BIN: BOLD:AAV7490; nearest neighbor: *Aleiodes* sp. BOLD:AAV6239, from French Guiana; distance to nearest neighbor is 8.65%. Consensus barcode. AATTTTATATTTTTTATTTGGTTTATGGTCAGGAATAATTGGCATGTCAATAAGATTAATTATTCGATTAGAATTAAGAACGAGAGGTAGAATTTTAAAAAATGACCAAATTTATAATGGCATAGTAACTTTACATGCATTTATTATAATTTTTTTTATAGTAATACCAATTATAATTGGTGGGTTTGGAAATTGATTAATTCCTTTAATATTAGGAGCCCCTGATATAGCATTTCCTCGTATAAATAATATAAGATTTTGATTATTAATCCCATCACTAATATTTTTATTGATTAGAGGTATTATTAATACAGGAGTAGGGACAGGATGAACTATATATCCTCCCCTATCTTCCTTAATTGGCCATAATAGAATATCAGTTGATATATCAATTTTTTCTCTCCATATAGCTGGAGCCTCATCAATCATAGGAGCAATTAATTTCATCTCAACAATTTTTAACATAAATCTAATAAAAATTAAAATAGACCAAATTATACTATTAGTATGGTCAGTTTTAATTACAGCTATTTTATTACTACTTTCATTACCTGTTTTAGCAGGAGCAATTACAATATTATTAACTGACCGTAATTTAAATACAAGATTTTTTGATTTTTCAGGAGGAGGGGACCCCATTTTATTCCAACATTTATTT.

##### Morphological data.

This species can be morphologically distinguished from its nearest neighbor by its uniformly colored hind legs (Fig. 31), compared to hind femur darker than remaining leg segments in the nearest neighbor.

**Figure 31.**
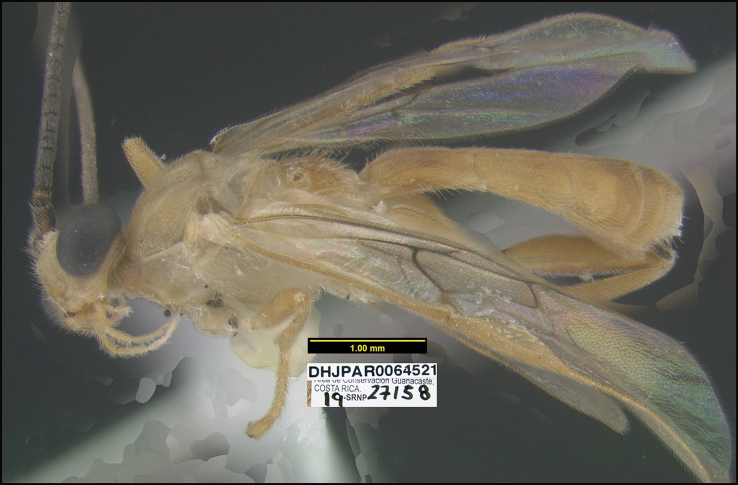
*Aleiodesjuniorporrasi*, holotype.

**Holotype** ?: Costa Rica: Alajuela, Guanacaste Area de Conservación, Sector Rincon Rain Forest, Sendero Aura, 432 m, 10.9654 -85.3239; host caterpillar collection date: 04/vii/2019, parasitoid eclosion: 31/vii/2019; depository CNC, holotype voucher code: DHJPAR0064521, GenBank accession: MW627570.

##### Holotype host data.

geoJanzen01 Janzen7158 (Geometridae) feeding on *Serjaniaschiedeana* (Sapindaceae), caterpillar voucher code:19-SRNP-27158.

##### Paratype.

BCLDQ01511, Honduras, Malaise-trapped (CNC).

##### Etymology.

*Aleiodesjuniorporrasi* is named in honor of Junior Porras Quirós, the BioAlfa Malaise traps manager for Estación Altamira, Parque Nacional Chirripo, ACLAP, Costa Rica.

#### ? Aleiodes rocioecheverri

Taxon classificationAnimaliaHymenopteraBraconidae

Sharkey sp. nov.

55F61DB3-C558-584F-8E23-B05A51A872EB

http://zoobank.org/E8FBEA44-9DE3-4E93-81F8-0CBAD405DB42

##### Diagnostics.

Figure 32.

##### BOLD data.

BIN: BOLD:AAM5673; nearest neighbor: *Aleiodes* sp. BOLD:AAH8820; distance to nearest neighbor is 4.03%. Consensus barcode: GTTTTATATTTTTTATTTGGGATATGAGCTGGTATATTAGGRTTATCTATAAGGTTAGTTATYCGTTTAGAATTAAGAAYTGTTGGRAGAGTTTTAAAAAATGATCAAATTTATAATGGKATGGTTACATTACATGCTTTTGTAATAATYTTTTTTATAGTTATACCTATTATAATTGGTGGGTTTGGAAATTGATTAATTCCTTTAATATTAGGGGCTCCTGATATAGCATTYCCTCGGATAAATAATATGAGATTTTGRTTATTAATTCCTTCATTTTTTTTATTATTAATTAGAGGTGTTATTAATTCAGGGGTAGGTACAGGTTGAACAATATACCCTCCCCTTTCTTTATTAATTGGTCATAATGGTTTATCAGTGGATATATCTATTTTTTCTTTACATTTAGCTGGRGCTTCTTCTATTATAGGATCAATTAATTTTATTTCAACTATTTTTAATATAAATTTATTTTATATTAAATTAGATCAGATTTCTTTATTAGTATGGTCAGTATTAATCACTACTATTTTATTATTATTATCTTTACCTGTTTTRGCAGGGGCTATTACTATATTATTGACTGATCGTAATTTAAATACAAGATTTTTTGATTTTTCGGGGGGAGGGGATCCAATTTTATTTCAACATTTA.

##### Morphological data.

There is no image on BOLD, but the p-distance makes it doubtful that it is conspecific.

**Holotype** ?: Costa Rica: Guanacaste, Area de Conservación Guanacaste, Sector San Cristobal, Estación San Gerardo, 575 m, 10.88 -85.389, 18/xi/2013, Malaise trap, depository CNC, holotype voucher code: BIOUG20202-G10, GenBank accession: MW627543.

**Figure 32.**
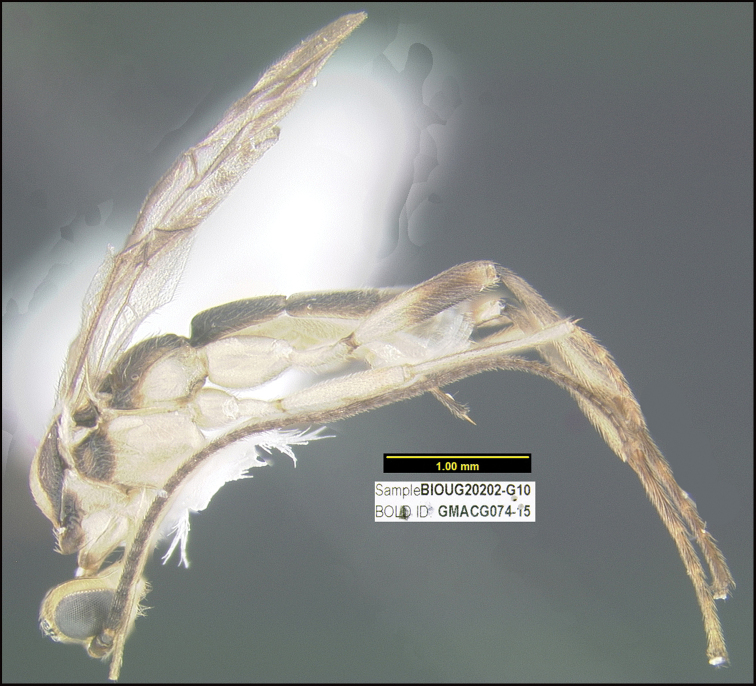
*Aleiodesrocioecheverri*, holotype.

**Other material.** BMNHE897799, from Belize deposited in the Natural History Museum (London), based on barcode, not viewed and lacking image on BOLD.

##### Etymology.

*Aleiodesrocioecheverri* is named in honor of Rocio Echeverri of San Jose and Liberia, Costa Rica, in recognition of her lifetime of concern and involvement with the betterment of Costa Rica’s positive relationship with its wild environment.

#### ? Aleiodes ronaldzunigai

Taxon classificationAnimaliaHymenopteraBraconidae

Sharkey sp. nov.

01BDD77E-547F-5861-BF97-A6940D4496B8

http://zoobank.org/A73F3D24-4F8D-42C3-94F3-EC56B5219C77

##### Diagnostics.

Figure 33.

##### BOLD data.

BIN: BOLD:AAH8707; nearest neighbor: Aleiodesnr.speciosusBOLD:AAM4342; distance to nearest neighbor is 2.4%. Consensus barcode: AATTTTATAYTTCATATTTGGAATATGAGCAGGTATAATTGGAATATCAATAAGATTAATTATTCGAATAGAATTAAGAACAAGAGGAAGAATTTTAAAAAATGACCAAATTTATAATGGTATAGTAACTTTGCATGCTTTTATTATAATTTTTTTTATAGTTATACCARTTATAATTGGGGGATTCGGAAATTGATTAATCCCCTTAATATTAGGGGCCCCTGATATAGCATTCCCTCGAATAAATAATATAAGATTTTGGTTATTAATTCCATCTTTATTATTTTTACTAATAAGAGGTATTATTAATACAGGAGTTGGGACAGGATGAACTATATACCCTCCTTTATCRTCTTTAATTGGACATAATAGAATTTCARTTGATATGTCAATTTTTTCTTTACATTTAGCAGGAGCTTCTTCTATTATAGGRGCAATTAATTTTATTTCAACAATTTTTAATATAAATTTAATAAAAATTAAATTAGACCAAATTTCATTGTTAATTTGATCAATTTTAATTACTACTATTTTATTATTATTATCTTTACCTGTACTAGCAGGAGCAATCACCATATTATTAACTGATCGTAACTTAAACACAAGATTTTTTGATTTTTCTGGAGGAGGAGAYCCAATTTTATTTCAACATTTATTT.

##### Morphological data.

No images are available on BOLD for the three specimens in the nearest neighbor, all from Ecuador.

**Holotype** ?: Costa Rica: Guanacaste, Area de Conservación Guanacaste, Sector Del Oro, Sendero Puertas, 400 m, 11.01087 -85.48816; host caterpillar collection date: 28/xii/2018, parasitoid eclosion: 07/i/2019; depository CNC, holotype voucher code: DHJPAR0064524, GenBank accession: MW627585.

**Figure 33.**
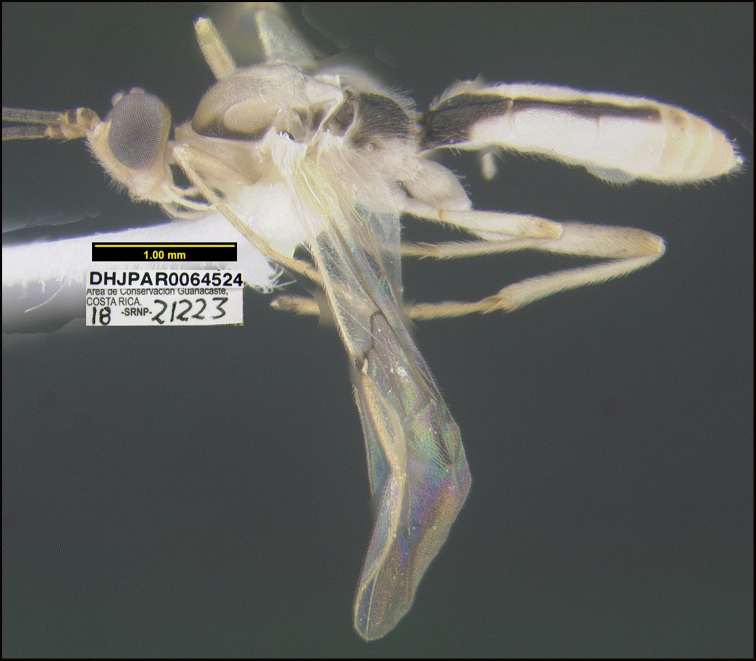
*Aleiodesronaldzunigai*, holotype.

##### Holotype host data.

geoJanzen01 19-SRNP-20029 (Geometridae) feeding on algae, caterpillar voucher code:18-SRNP-21223.

##### Paratype.

all males and all with same host as holotype, DHJPAR0064525, DHJPAR0064526, DHJPAR0063992.

##### Etymology.

*Aleiodesronaldzunigai* is named in honor of Ronald Zúñiga, the BioAlfa Malaise traps manager for Parque Ecológico, SINAC, Santo Domingo de Heredia, ACC (Area de Conservación Central), Costa Rica.

#### ? Choreborogas jesseausubeli

Taxon classificationAnimaliaHymenopteraBraconidae

Sharkey sp. nov.

236166A3-241E-5A99-9034-2FAAC25B4297

http://zoobank.org/20C776D0-74D9-4B3D-B0E0-EE4E117B28CB

##### Diagnostics.

Figure 34.

##### BOLD data.

BIN: BOLD:AAM5951; nearest neighbor: *Choreborogas* sp. BOLD:ACG8400; distance to nearest neighbor is 2.71%. Consensus barcode:

AGTATTGTATTTTTTTTTTGGTATATGATCAGGTATATTGGGYTTATCAATAAGGTTAATTATTCGGTTTGAATTAGGGGTTCCTGGATCATTTTTAGGTAATGATCAGATTTATAATAGAATTGTTACGGCYCATGCCTTGGTTATAATTTTTTTTATGGTTATACCTGTAATAATTGGGGGATTTGGTAATTGATTAATTCCTTTAATATTAGGRGCACCTGATATAGCTTTYCCTCGAATAAATAATATAAGATTTTGGTTATTAATTCCTTCTATTTTGTTATTGTTAGTTAGATCTTTAGTTAATGTTGGGGYAGGTACAGGATGAACAATTTATCCTCCTTTATCTTCRTTAATAGGTCATGGSGGGATTTCAGTTGATTTAGCTATTTTTTCTTTACATTTAGCTGGTGCATCATCAATTATAGGTGCAATTAATTTTATTTCTACAATTTTTAATATAAATTTATTTTCAATGAAAATAGATCAAATTATATTATTAGTTTGATCTGTATTAATCACTGCTTTTTTATTATTATTATCATTRCCTGTTTTGGCGGGGGCAATTACTATATTATTATTTGATCGTAAYATTAATAGAACTTTTTTTGATTTTTCAGGGGGAGGGGATCCTATTTTATTYCAGCATTTATT

##### Morphological data.

This species can be morphologically distinguished from its nearest neighbor by its swollen hind basitarsus (Fig. 34), which is much narrower in the nearest neighbor. Males lack the swollen hind femora.

**Figure 34.**
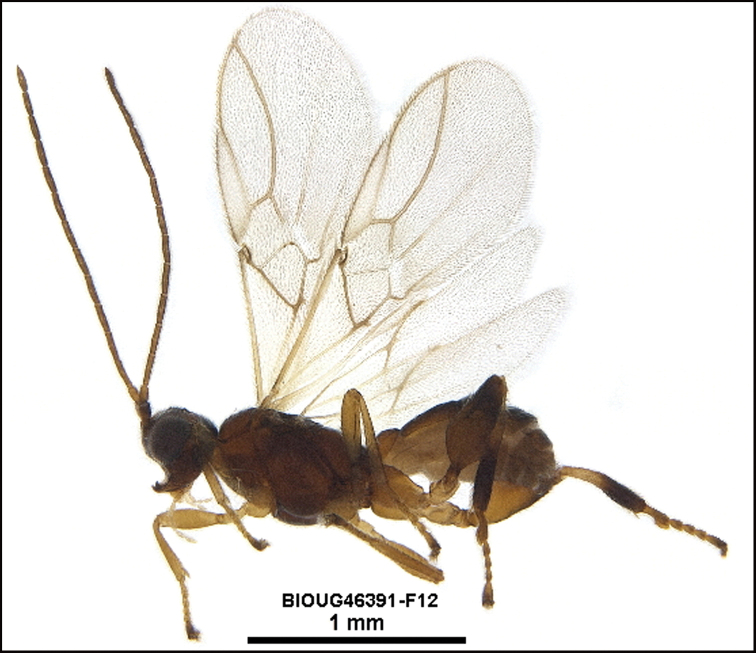
*Choreborogasjesseausubeli*, holotype.

**Holotype** ?: Costa Rica: Guanacaste, Area de Conservación Guanacaste, Sector Pailas Dos, PL12-6, 853 m, 10.7637 -85.3331, 04/xii/2014, Malaise trap, depository CNC, holotype voucher code: BIOUG46391-F12, GenBank accession: MW627542.

##### Paratype.

Malaise trapped, BIOUG46544-F12, BIOUG49790-H06, BIOUG07453-F05, BIOUG28810-A07, BIOUG29020-B09.

**Other material**: BMNHE897774 from Belize is in the same BIN and likely conspecific. There is no image on BOLD and the specimen was not examined.

##### Etymology.

*Choreborogasjesseausubeli* is named in honor of Jesse Ausubel of Rockerfeller University, New York, USA, for his very strong support of the germination and early development of DNA barcoding as an identification tool.

#### ? Triraphis doncombi

Taxon classificationAnimaliaHymenopteraBraconidae

Sharkey sp. nov.

BC1796D0-995C-5151-89B9-479740A8C5D5

http://zoobank.org/8228960E-CF6E-4BF9-8C86-696F7EA67FE5

##### Diagnostics.

Figures 35–37.

##### BOLD data.

BIN: BOLD:AAH8815; nearest neighbor: *Triraphis* sp. BOLD:AAG5003 from Guyana. Distance to nearest neighbor is 6.28%. Consensus barcode:TGTTTTATATTTTTTATTTGGAATTTGAGCTGGTATAGTCGGGCTGTCTATAAGGTTAATTATTCGGTTAGAATTAAGTATACCAGGGAGATTATTGGGGAATGAYCAGATTTATAATGGTATAGTTACCGCTCATGCTTTTATTATAATTTTTTTTATGGTAATACCTATTATAATTGGTGGTTTTGGAAATTGATTAATTCCATTAATGTTGGGGGCYCCTGATATGGCTTTCCCTCGTATAAATAATATGAGGTTTTGGTTATTAATTCCYTCATTGACGTTATTAATTTTAAGGGCTGTAGTTAACGTTGGAGTAGGTACTGGGTGAACTTTATATCCYCCCTTATCTTCTTTAGTTGGTCATGGGGGTATATCTGTAGATATAGCTATTTTTTCTTTACATTTAGCTGGTGCCTCTTCTATTATAGGAGTTGTTAATTTTATTTCTACTATTTTTAATATAAAATTAATATCAATTAATTTAGATCAAATTAATTTATTTGTTTGATCAGTATTAATTACGGCTGTTTTATTATTATTATCTTTACCAGTATTAGCTGGTGCAATTACTATATTATTGACAGATCGTAATTTAAATACAACATTTTTTGATTTTTCTGGGGGGGGYGATCCTATTTTATTCCAACATTTATTT

##### Morphological data.

No image of the nearest neighbor is available on BOLD, but the COI distance and geographic distribution suggest that they are not the same species.

**Holotype** ?: Costa Rica: Guanacaste, Area de Conservación Guanacaste, Sector Pitilla, Sendero Naciente, 700 m, 10.98705 -85.42816; host caterpillar collection date: 09/ii/2010, parasitoid eclosion: 13/ii/2010; depository CNC, holotype voucher code: DHJPAR0038023. GenBank accession: HQ548697.

**Holotype host data.***Eucleamesoamericana* (Limacodidae) feeding on *Thelypterisnicaraguensis* (Thelypteridaceae), caterpillar voucher code: 10-SRNP-30444.

**Figure 35.**
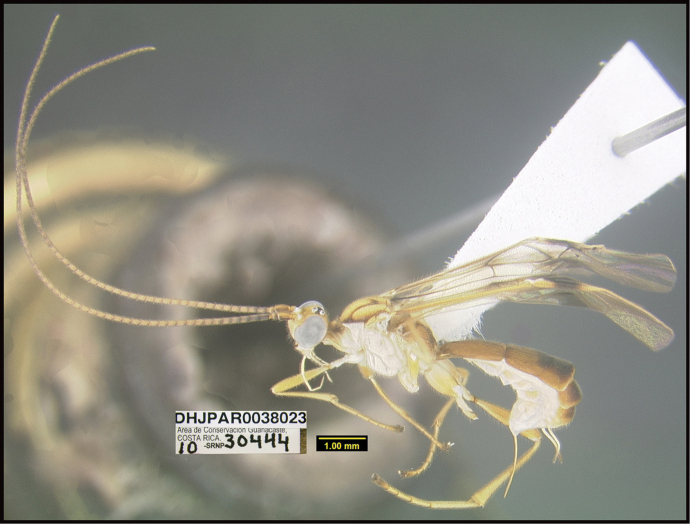
*Triraphisdoncombi*, holotype.

**Figure 36.**
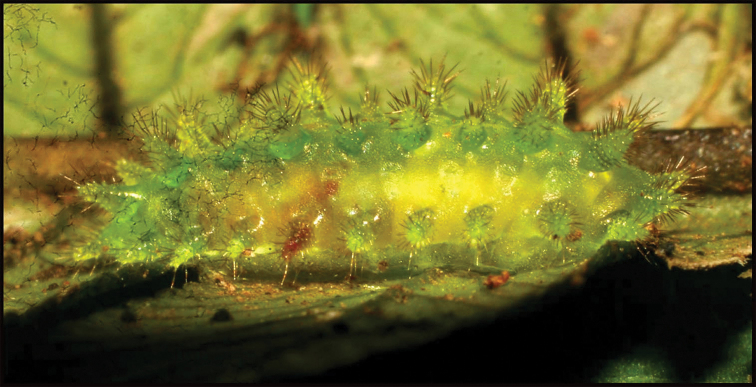
White pupa of wasp *Triraphisdoncombi* (DHJPAR0038023) visible through the translucent body wall of the parasitized host caterpillar *Eucleamesoamericana* (Limacodidae) in its last instar.

**Figure 37.**
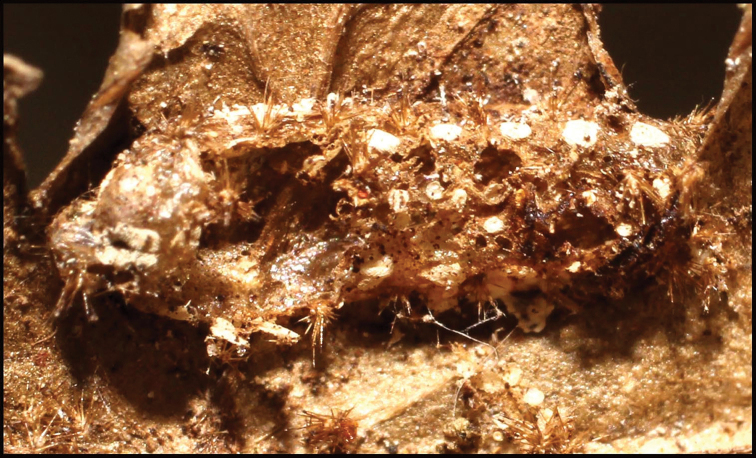
Exit hole, left side, cut by the wasp *Triraphisdoncombi* (DHJPAR0038023) to exit the mummified body wall of the parasitized host caterpillar *Eucleamesoamericana* (Limacodidae) in its last instar.

##### Paratype.

BCLDQ0860.

##### Etymology.

*Triraphisdoncombi* is named in honor of Dr. Don Comb (RIP), founder of the New England Biolabs and New England Biolabs Foundation, in recognition of his serious and ongoing support for the management and biodiversity conservation of Área de Conservación Guanacaste in northeastern Costa Rica (http://www.acguanacaste.ac.cr), through the Guanacaste Dry Forest Conservation Fund (http://www.acguanacaste.ac.cr).

#### ? Yelicones mayrabonillae

Taxon classificationAnimaliaHymenopteraBraconidae

Sharkey sp. nov.

92179AC2-B0F2-536F-A9EC-A5CE15FADB3F

http://zoobank.org/8BE983A2-E1D9-4FB8-AC3C-6E2FB4B5F8AA

##### Diagnostics.

Figures 38, 39.

##### BOLD data.

BIN: BOLD:AAT9450. Its nearest neighbor on BOLD is identified by D. Quicke as *Yeliconesartitus*BOLD:AAM6954; distance to nearest neighbor is 9.2%. Consensus barcode: TGTTTTATATTTTTTATTAGGTATTTGATGTGGATTAGTAGGTTTATCTTTAAGGTTACTTATTCGGTTAGAGTTGAGAAATTTAGGAAGATTATTAGGTAATGATCAAATTTATAATGTAGTTGTTACTATACATGCTTTTATTATAATTTTTTTTATGGTGATACCTATTATAATTGGAGGTTTTGGAAATTGATTAATTCCCTTAATATTAGGAGCTCCTGATATAGCTTTCCCTCGTATAAATAATATAAGATTTTGATTATTAATTCCTTCTTTGTTATTAATATTAATAAGAGGATTTATTATAGTTGGTAGTGGAACTGGGTGAACTATATATCCTCCTTTAAGTTCTTTAATTGGGCATAGAAGGTTTTCTGTTGATATAGTAATTTTTTCTTTACATTTAGCAGGGGTTTCTTCAATTATAGGAGCTATTAATTTTATTACAACAATTTTTAATATAAAATTAATTT---TAAAATTAGATCAGATTATATTATTTGTATGATCTGTATTAATTACTGCTTTTTTATTATTACTTTCTTTACCTGTTTTGGCAGGAGGAATTACTATATTATTAACAGATCGTAATTTAAATACTTCTTTTTTTGATTTTTCAGGAGGGGGAGATCCTGTTTTATTTCAACACTTATTT.

##### Morphological data.

This species keys to *Y.vilawanae* in the key of Areekul and Quicke (2006). *Yeliconesvilawanae* has the apical 0.2 of hind tarsus and apical 0.8 of hind basitarsus brown. *Yeliconesmayrabonillae* has the basal four hind tarsomeres brown and the apical tarsomere yellow. This species can be morphologically distinguished from its nearest neighbor, *Yeliconesartitus*, by the color of the hind femur being entirely testaceous (Fig. 38) (apical .04 brown in *Y.artitus*).

**Figure 38.**
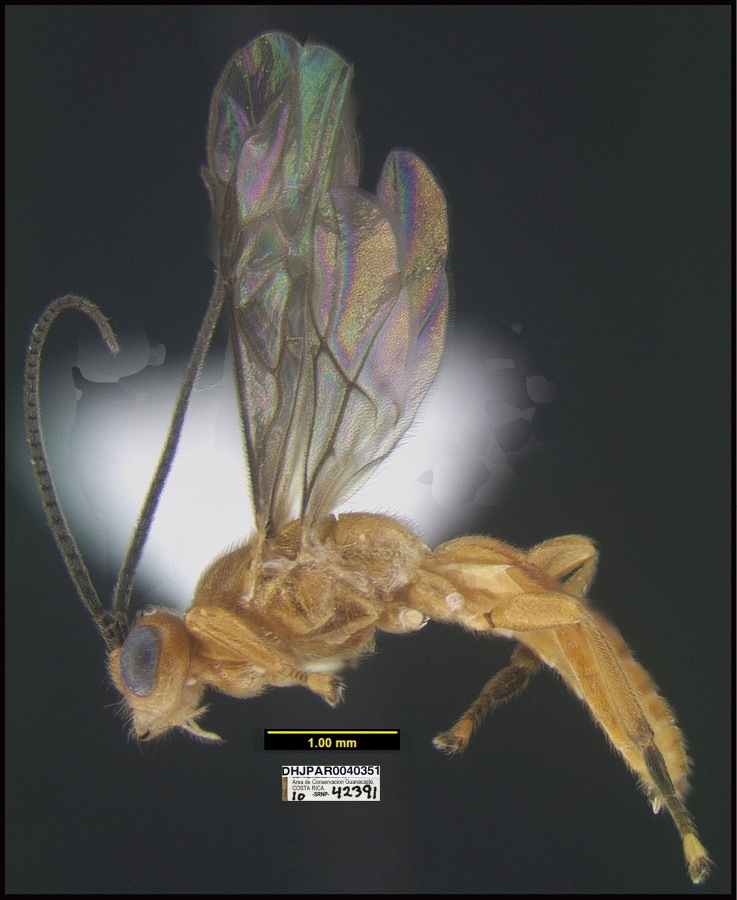
*Yeliconesmayrabonillae*, holotype.

**Figure 39.**
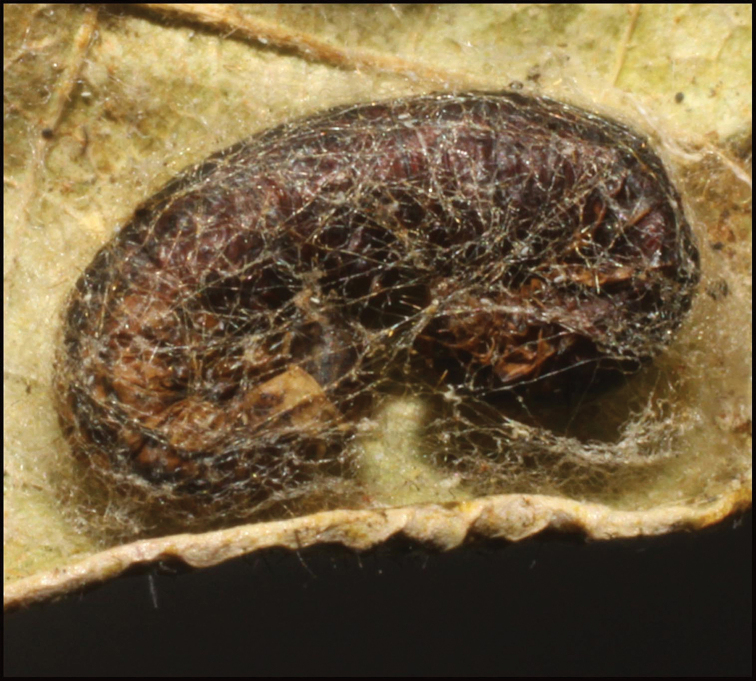
*Yeliconesmayrabonillae*, remains of host caterpillar, epipajanzen01 Janzen882 (Pyralidae); note mummified host caterpillar curved into a distinctive “C” shape, characteristic of other species of pyralid caterpillars attacked by species of *Yelicones*.

**Holotype** ?: Costa Rica: Alajuela, Area de Conservación Guanacaste, Sector Rincon Rain Forest, Sendero Venado, 420 m, 10.897 -85.27; host caterpillar collection date: 26/vi/2010, parasitoid eclosion: 03/viii/2010; depository CNC, holotype voucher code: DHJPAR0040351.

##### Holotype host data.

epipajanzen01 Janzen882 (Pyralidae) feeding on *Vochysiaguatemalensis* (Vochysiaceae), caterpillar voucher code:10-SRNP-42391.

##### Etymology.

*Yeliconesmayrabonillae* is named in honor of Mayra Bonilla as the primary supporter of the BioAlfa Malaise trapping at the Hacienda Baru Wildlife Refuge, Savegre, ACOPAC, Costa Rica, as well as decades of support for the Area de Conservación Guanacaste.

## Supplementary Material

XML Treatment for Aerophilus davidwagneri

XML Treatment for Aerophilus fundacionbandorum

XML Treatment for Aerophilus nicklaphami

XML Treatment for Amputoearinus alafumidus

XML Treatment for Lytopylus davidstopaki

XML Treatment for Lytopylus davidschindeli

XML Treatment for Gnathopleura josequesadai

XML Treatment for Bracon andreamezae

XML Treatment for Bracon franklinpaniaguai

XML Treatment for Bracon rafagutierrezi

XML Treatment for Bracon guillermoblancoi

XML Treatment for Bracon oscarmasisi

XML Treatment for Bracon pauldimaurai

XML Treatment for Bracon shebadimaurae

XML Treatment for Sacirema karendimaurae

XML Treatment for Chelonus minorzunigai

XML Treatment for Homolobus stevestroudi

XML Treatment for Macrocentrus michaelstroudi

XML Treatment for Stantonia gilbertfuentesi

XML Treatment for Rhysipolis stevearonsoni

XML Treatment for Aleiodes kaydodgeae

XML Treatment for Aleiodes kerrydresslerae

XML Treatment for Aleiodes josesolanoi

XML Treatment for Aleiodes juniorporrasi

XML Treatment for Aleiodes rocioecheverri

XML Treatment for Aleiodes ronaldzunigai

XML Treatment for Choreborogas jesseausubeli

XML Treatment for Triraphis doncombi

XML Treatment for Yelicones mayrabonillae
